# ATH-1105 mitigates multiple pathologies in ALS models both alone and in combination with riluzole

**DOI:** 10.3389/fneur.2025.1582765

**Published:** 2026-01-08

**Authors:** Andrée-Anne Berthiaume, Kayla N. Kleist, Sherif M. Reda, Sharay E. Setti, Wei Wu, Jewel L. Johnston, Robert W. Taylor, Liana R. Stein, Kevin J. Church

**Affiliations:** Athira Pharma, Inc., Bothell, WA, United States

**Keywords:** ALS, hepatocyte growth factor, neurotrophic factor, neurofilament light chain, TDP-43, neuroprotection, small-molecule therapeutics, riluzole

## Abstract

**Introduction:**

Amyotrophic lateral sclerosis (ALS) is a fatal neurodegenerative disorder characterized by progressive motor neuron degeneration, muscle atrophy, and paralysis. The complexity of ALS pathology, driven by factors such as TDP-43 pathology, excitotoxicity, and neuroinflammation, has hindered therapeutic development. While riluzole (an anti-excitotoxic agent) is the current standard treatment, additional therapeutics are needed to address the broad spectrum of ALS-related pathology. ATH-1105, a small-molecule positive modulator of hepatocyte growth factor (HGF) signaling, has shown promise in preclinical models of ALS. Given the multifactorial nature of ALS and the growing recognition that combination approaches may represent the best treatment options, we investigated the therapeutic potential of ATH-1105 in a TDP-43-driven mouse model of ALS, by comparing and combining it with the known efficacious treatment of riluzole. Additionally, we characterize the mechanism by which ATH-1105 induces neuroprotective effects, emphasizing its effects on TDP-43 pathology.

**Methods:**

*In vivo*, the impact of daily oral treatment with ATH-1105, alone and in combination with riluzole, was evaluated in Prp-TDP43^A315T^ hemizygous transgenic ALS mice. *In vitro*, the impact of ATH-1105 on TDP-43-related pathology was assessed in rat primary spinal motor neurons subjected to glutamate toxicity. To demonstrate target engagement, the neuroprotective effects of ATH-1105 were assessed via siRNA-mediated knockdown of MET (HGF receptor).

**Results:**

*In vivo*, ATH-1105 significantly improved neuromuscular function and reduced body weight loss, neurodegeneration, inflammation, and TDP-43 phosphorylation. The combination of ATH-1105 with riluzole led to greater therapeutic effects than either treatment alone. *In vitro*, the neuroprotective effects of ATH-1105 were shown to be associated with MET activation in motor neurons, which was confirmed via siRNA-mediated knockdown of MET. In motor neurons subjected to glutamate toxicity, ATH-1105 reduced extranuclear and phosphorylated TDP-43, and increased GSK3β phosphorylation (inactivation), a kinase involved in TDP-43 pathology. Additionally, ATH-1105 reduced the abnormal increase in autophagic proteins following glutamate toxicity.

**Discussion:**

Our study underscores the therapeutic potential of ATH-1105 in treating ALS, both as a standalone treatment and in combination with riluzole. ATH-1105 demonstrates neuroprotective effects that slow neuromuscular deterioration in a relevant mouse model, aligning with the need to counteract the neurodegeneration central to ALS.

## Introduction

Amyotrophic lateral sclerosis (ALS) is the most prevalent motor neuron disease in adults. It is a fatal condition characterized by progressive motor neuron degeneration in the brain and spinal cord, leading to muscle weakness and atrophy, motor impairment, and ultimately, paralysis and respiratory failure. Due to its rapid progression and devastating prognosis, the average survival rate is just 2 to 5 years from symptom onset, highlighting the critical unmet medical need to address disease progression in people living with ALS.

Identifying new treatments for ALS has been challenging due to the disease’s complex nature, driven by multiple pathological mechanisms rather than a single event. Indeed, protein pathology, mitochondrial dysfunction, oxidative stress, neuroinflammation, autophagic impairment, excitotoxicity, and neuromuscular junction (NMJ) dysfunction have all been implicated in the pathogenesis of the disease. Among these, TAR DNA-binding protein 43 (TDP-43) protein pathology is notably prevalent, affecting 97% of people with ALS (1, 2). TDP-43 is a DNA/RNA binding protein that is mainly located in the nucleus where it regulates critical functions of RNA metabolism. In ALS, TDP-43 translocates to the cytoplasm, forming toxic inclusions and interfering with normal cellular processes (2). However, what initially precipitates the nuclear depletion and extranuclear accumulation of TDP-43 remains unclear. Leading hypotheses suggest that abnormal post-translational modifications, such as kinase-mediated phosphorylation, ubiquitination, and fragmentation, may contribute to TDP-43 aggregation and its impaired re-entry into the nucleus (3, 4). Another potential mechanism that has been suggested involves defects in the nuclear pore complex, which normally regulates the proper shuttling of TDP-43 in and out of the nucleus (5). Furthermore, impaired cellular autophagy may also exacerbate TDP-43 accumulation by reducing the ability of neurons to properly clear abnormal proteins effectively (6, 7). Regardless of the initiating factors, the presence of extranuclear TDP-43 in the vast majority of clinical ALS cases points to this as a critical disease component, and its reduction would likely prove beneficial. Although targeting TDP-43 pathology may be promising, this alone may not be sufficient as a comprehensive therapeutic strategy. As previously mentioned, glutamate excitotoxicity, mitochondrial dysfunction, oxidative and cellular stress, and neuroinflammation have also all been linked to the disease and play significant roles, both individually and collectively, in its progression (3, 8, 9). It is likely the summation of these components, along with TDP-43 dysfunction, that ultimately drives motor neuron degeneration.

Perhaps partially attributed to the complex pathology described above, few therapeutics have received FDA approval for the treatment of ALS. The most prescribed medication is riluzole, which received approval in 1995 (10). Riluzole is thought to slow disease progression by reducing glutamatergic activity, thereby mitigating excitotoxic neuronal injury. Riluzole has been shown to modestly prolong tracheostomy-free survival by an average of 3 months, although more recent studies suggest that the median survival may be substantially longer (11). Until recently, it remained the only pharmaceutical intervention that had any positive clinical effect on the disease and has become the standard of care. Other therapeutic options include edaravone and tofersen. Edaravone, an antioxidative agent thought to reduce the burden of oxidative stress in motor neurons (12), has demonstrated mixed outcomes in recent clinical trials and as such has not been adopted broadly (13, 14). Tofersen, an antisense oligonucleotide targeting superoxide dismutase 1 (SOD1), was granted accelerated approval by the FDA in 2023 for the 2–3% of familial ALS patients carrying this genetic mutation. Tofersen demonstrated effects on neurodegeneration via the fluid biomarker neurofilament light chain (NfL) in a clinical trial, as well as a pattern of improved function seen during the open label extension (15). Despite these recent efforts and advances, there remains a critical unmet need towards addressing the more highly represented non-SOD1 and sporadic forms of the disease.

In this context, ATH-1105 emerges as a promising candidate with the ability to simultaneously address multiple pathologies associated with ALS. ATH-1105 is an orally bioavailable, blood–brain barrier penetrant, small molecule positive modulator of the neurotrophic hepatocyte growth factor (HGF) signaling system, acting through its sole receptor MET. A comprehensive assessment of ATH-1105’s multimodal effects in preclinical ALS models, previously published by our group, demonstrates its potent neuroprotective and anti-inflammatory effects targeting multiple key mechanisms in ALS (16). ATH-1105 was reported to protect motor neurons against glutamate-induced toxicity and associated pathological changes, including mitochondrial dysfunction, apoptotic signaling, astrocyte reactivity, NMJ abnormalities, and motor neuron degeneration. Previous reports also include preliminary observations of the potential impact of ATH-1105 treatment on TDP-43 pathology (16).

The goals of the current work were three-fold: (1) to expand on our understanding of the therapeutic potential of ATH-1105 in a TDP-43 driven mouse model of ALS by comparing and combining it with the known efficacious treatment riluzole, (2) to understand the effect of ATH-1105 on some of the cellular mechanisms associated with TDP-43 pathology, and (3) to verify that the mechanism of action of ATH-1105 is dependent on MET signaling in motor neurons, as evidence of target engagement *in vitro*. Our findings support the continued development of ATH-1105 for the treatment of ALS. In a Phase I, double-blind, placebo-controlled trial in healthy volunteers, ATH-1105 demonstrated a favorable safety and tolerability profile, dose-proportional PK, and CNS penetration (NCT06432647).

## Methods

### Animals

*In vivo* studies were conducted using male Prp-TDP43^A315T^ (ALS) mice (Jackson Laboratory Strain #:010700) and age-matched wild-type (WT) counterparts following previously published protocols (16). Briefly, these ALS mice express mutant TDP-43 cDNA with an alanine-to-threonine substitution at the 315th amino acid (A315T), a mutation linked to familial ALS (17). Animals were housed in Markrolon cages (Innovive, M-BTM) with filter hoods to prevent contamination. Water and nutrition was provided ad libitum, and a jellified diet was provided to mitigate gastrointestinal dysmotility and malnutrition (18). Environmental conditions were controlled, maintaining a constant temperature and a 12 h light/dark cycle. The experiments were carried out in Lunel, France, by In Vivex SAS, under a protocol approved by the Animal Studies Committee of Languedoc Roussillon (reference No.: D3417223, APAFIS#23920–2020020320279696 v3). Animal health was monitored daily.

This study was performed using 50 male mice, divided into 5 groups with 10 mice per group. Animals were randomized into groups based on body weight, and body weight was measured weekly upon the initiation of treatment. Each mouse received its respective treatment from 1 to 3 months of age, for a total of 2 months of daily treatment. The groups were as follows: WT + vehicle, ALS + vehicle, ALS + ATH-1105 20 mg/kg, ALS + Riluzole 5 mg/kg, ALS + ATH-1105 20 mg/kg + Riluzole 5 mg/kg. The vehicle consisted of 2% dimethyl sulfoxide (DMSO), 20% PEG-400, and 78% saline solution. ATH-1105 and associated vehicle control was administered orally (p.o.), while riluzole and associated vehicle control was delivered intraperitoneally (i.p.) and formulated in saline. Experimenters were blind to treatment.

### Plasma exposure of ATH-1105 and riluzole

On the final day of the two-month treatment period, blood samples were collected from the tail vein of all animals 60 min after treatment administration into tubes containing ethylenediaminetetraacetic acid (EDTA) as an anticoagulant. Plasma was separated by centrifugation then stored at −80 °C until analysis. The concentrations of ATH-1105 and riluzole in plasma was determined by high performance liquid chromatography coupled with tandem mass spectrometry (HPLC-MS/MS). Bioanalysis was performed by Eurofins (Vergèze, France). Calibration curves were generated with plasma standards ranging from 1 to 2000 ng/mL. Quantification was performed using peak area ratios of the analyte to the internal standard using Watson^®^ 7.5 SP1. Automatic chromatogram integration was conducted using Analyst^®^ 1.6.3 or LabSolutions V6.60 software. Concentrations below the lower limit of quantification were considered as zero for calculations, and the measured concentrations were averaged per group.

### Motor function tests

Behavioral assessments were performed monthly during the light cycle. Tests included balance beam, rotarod, grip strength, and Kondziela screen. The balance beam test was used to assess balance and coordination. Mice were timed as they crossed a narrow beam (3 cm in circumference) that was elevated 50 cm above the ground. The average of three trials per day conducted at 5 min intervals for each animal was recorded.

Walking performance, balance, and coordination were assessed using a rotarod apparatus. In each trial, the rotation speed was gradually increased from 4 to 40 rpm over 300 s, the maximal trial length. Animals were pretrained on the apparatus the day prior to testing. On the day of testing, latency to fall from the rotating platform was recorded and averaged across three trials.

The grip strength test evaluated neuromuscular strength by measuring the maximum force (in Newtons) exerted by all four limbs gripping a horizontal wire grid while the experimenter pulled the animals tail parallel to the grid. This force was averaged across three trials.

Lastly, muscular strength and proprioception was measured using the Kondziela screen test. Animals were placed at the top of a vertical wire grid and allowed to climb downward. The latency to fall from the grid was recorded and averaged across three trials.

Motor performance was evaluated as both the absolute value at each time point and the change from pretreatment baseline to end of study after 2 months of treatment (CFB).

### Sciatic nerve electrophysiology

Standard electromyography was conducted monthly on mice anesthetized with a ketamine-xylazine mixture. A pair of steel-needle electrodes (AD Instruments, Sydney, Australia; MLA1302) were placed subcutaneously at the sciatic notch, and a second pair was placed along the tibial nerve above the ankle. Supramaximal square wave pulses (10 ms at 1 mA) were delivered using a PowerLab 26 T (AD Instruments). The amplitude and latency of the compound muscled action potential (CMAP) was measured from the intrinsic foot muscles. The distance between the two stimulation sites was measured along the skin surface while the leg was fully extended. This measurement, combined with the latency data, was used to calculate the nerve conduction velocity (NCV). CMAP and NCV were evaluated over time and as a CFB.

### Plasma biomarkers

Plasma interleukin-6 (IL-6), tumor necrosis factor alpha (TNF-*α*), and NfL levels were quantified using enzyme-linked immunosorbent assay (ELISA). Blood was collected from the submandibular vein into microtubes containing EDTA. Plasma was obtained via centrifugation and stored at −20 °C until analysis. Plasma samples were diluted 1:10 in sterile phosphate-buffered saline (PBS). Levels of IL-6, TNF-*α*, and NfL were measured in duplicate using ELISA kits from Sigma-Aldrich (IL-6: RAB0308, TNF-α: RAB0477) and Novus Biologicals (NfL: NBP2-80299).

### Sciatic nerve TDP-43

For the assessment of phospho-TDP-43, the right sciatic nerve was collected and flash-frozen in liquid nitrogen. The frozen tissue was homogenized using sonication in sterile PBS on ice followed by centrifugation at 10,000 rpm for 10 min at 4 °C. Homogenized samples were tested using the Amplified Luminescent Proximity Homogenous Assay (AlphaLISA) *SureFire* Human Phospho-TDP-43 ELISA Kit (PerkinElmer, ALSU-PTDP43) according to the manufacturer’s instructions. Each sample was tested in duplicate at 10 ng/mL total protein, verified by a NanoDrop spectrophotometer (Thermo Fisher Scientific). Phospho-TDP-43 quantification was carried out using a fluorescence microplate reader.

### Morphometric analysis of the sciatic nerve

Mice were sacrificed by cervical dislocation followed by exsanguination through decapitation. The right sciatic nerve was fixed *in situ* for 2 min using 4% glutaraldehyde. Afterward, a 1.5 cm segment of the sciatic nerve was excised and fixed overnight and stored in 4% glutaraldehyde at 4 °C until further processing.

Histological analysis was performed at NeuroFit S.A.S (Illkirch, France). Nerve samples were rinsed with PBS at room temperature (RT) (2 × 10 min) and post-fixed in 1% osmium tetroxide (EMS ref. 1919-lot 110,512) at RT for 3 h, then rinsed in PBS at RT (2 × 10 min). To prepare the samples for embedding, a series of graded alcohol dehydration steps were carried out as follows: 50% alcohol for 10 min, 60% alcohol for 10 min, 70% alcohol overnight at RT, 80% alcohol for 10 min, 90% alcohol for 10 min, 95% alcohol for two 10 min sessions, and 100% alcohol for three rounds of 1 h each.

The dehydrated tissues were then embedded in Epon. The preparation of Epon involved combining Embed 812 (Electron Microscopy Sciences, 14,900) and dodecenyl succinic anhydride (DDSA; Electron Microscopy Sciences, 13,710) for Epon A (31 parts Embed 812 + 50 parts DDSA) and Embed 812 and nadic methyl anhydride (NMA; Electron Microscopy Sciences, 19000) for Epon B (10 parts Embed 812 + 9 parts NMA). Tissues were placed in an Epon/alcohol mixture (Epon A: 1 part, Epon B: 1 part, 100% alcohol: 2 parts) for 3 h. The samples were then left overnight in an Epon solution (Epon A: 1 part, Epon B: 1 part) at RT. A further incubation was performed in an inclusion medium for 1 h at RT (Epon A: 1 part, Epon B: 1 part, 2,4,6-tridimethylaminomethyl phenol (DMP-30; Electron Microscopy Sciences, 13600)) at a volume of 1.7% of the combined Epon A + B mixture. The embedded samples were then subjected to a polymerization process at 70 °C for a duration of 3 days.

Using a microtome (Microm HM360), the embedded tissue blocks were sectioned. Initially, transverse sections of 5 μm were cut until a suitable cross-section near the midpoint of the nerve segment was identified under a microscope. Subsequently, thinner sections of 1.5 μm were produced and stained with 1% toluidine blue (Electron Microscopy Sciences, 92319-CI 52040) for approximately 2 min. These sections were then rinsed three times in PBS for 10 min each, followed by serial dehydration in 95 and 100% alcohol. This was followed by a 10 min treatment in a xylene substitute solution (Electron Microscopy Sciences, 2341001) at RT. The sections were then mounted in Eukitt (Electron Microscopy Sciences, 15320).

To perform morphometric analysis, a composite image of each nerve section was captured using an optical microscope (Nikon/optiphot-2) paired with a digital camera (Nikon DS-Fi1). The acquired digital images were analyzed using Image-Pro Plus software (Media Cybernetics Inc., United States). This software measured axonal and myelin dimensions by isolating each myelinated fiber based on the gray intensity of the toluidine blue-stained myelin sheath.

The analysis involved two distinct segmentation processes. The first segmentation generated a binary image where axoplasms were highlighted against a dark background, enabling the determination of axonal properties such as size and distribution. Axon caliber was determined as the area of the axon expressed in square micrometers (μm^2^). The axon diameter and caliber were determined for each axon in a single cross section per animal. The second segmentation produced a binary image where the myelin sheaths appeared as dark rings surrounding a bright central area (axoplasm) on a bright background. These measurements were used to compute the g-ratio, an indicator of myelin sheath thickness relative to axon size, defined as the ratio of the axonal diameter to the fiber diameter, using the formula [A/(A + M)]^0.5^ (where A is the axonal area and M is the myelin area). These g-ratio values were then plotted against the corresponding axon calibers to generate regression lines for the g-ratio distribution.

### Statistical analyses for *in vivo* studies

Statistical analyses were conducted with GraphPad Prism software (version 10; GraphPad Software, Inc., San Diego, CA). Data is represented as mean ± SEM. Statistical significance of motor performance and electrophysiology over time was determined using two-way analysis of variance (ANOVA) with Dunnett’s multiple comparisons test under the assumptions of normal distribution and homoscedasticity. CFB analyses was assessed with a one-way ANOVA followed by Tukey’s multiple comparisons. Statistical significance of plasma biomarkers and sciatic nerve assessments (pTDP-43 and g-ratio/axon caliber slope) was assessed with two and one-way ANOVA, respectively, with Tukey’s multiple comparisons. *Post hoc* significance is reported only for comparisons where the ANOVA test indicated significance. Comparisons were considered statistically significant at *p* < 0.05.

### Primary motor neuron culture

Rat spinal cord motor neurons were cultured as described in Boussicault et al. (19). Briefly fetal Wistar rats at 14 days of gestation were removed from pregnant females. Spinal cords were removed and treated with trypsin–EDTA solution (0.05%, 20 min at 37 °C) and cells were mechanically dissociated by forced passage through the tip of a 10 mL pipette. Cells were then centrifuged at 515 x g for 10 min at 4 °C. The supernatant was discarded, and the pellet was resuspended in a defined culture medium consisting of Neurobasal medium with a 2% solution of B27 supplement, 2 mM of L-glutamine, 2% of penicillin–streptomycin (PS) solution, and 10 ng/mL of brain-derived neurotrophic factor (BDNF). The cells were seeded at a density of 20,000 per well in 96-well plates pre-coated with poly-L-lysine and were cultured at 37 °C with media changes every 2 days.

### Glutamate neurotoxicity assays

#### Immunofluorescence analysis: neuronal survival, neurite network, extranuclear TDP-43, and LC3

On culture day 13, test compounds were applied in vehicle (basal media, 0.1% DMSO, 0.05 ng/mL HGF) for 15 min. Without removal of test compound, glutamate (5 μM) was added to the culture for 20 min followed by wash-out and re-application of test compounds. Test compounds were refreshed every 24 h.

After 24 h in culture, cultures were fixed with 4% paraformaldehyde in PBS, pH 7.3 for 2 h at RT and then permeabilized with 0.1% saponin. Fixed permeabilized cultures were incubated with the following primary antibodies: chicken anti microtubule-associated-protein 2 (MAP2, 1:400) (Abcam; #Ab5392), rabbit anti microtubule-associated protein 1A/1B-light chain 3 (LC3b) (1:50) (Abcam; #Ab192890), and mouse anti-nuclear TDP-43 (1:1000) (Abcam; #Ab104223). Primary antibodies were revealed with Alexa Fluor 488 anti-mouse IgG (Sigma-Aldrich; #SAB4600042-250ul) at the dilution 1:400, Alexa Fluor 568 anti-rabbit IgG (Sigma-Aldrich; #SAB4600084-250ul) at the dilution 1:400 and with CF™ 647 anti-chicken IgG (Sigma-Aldrich; #SAB4600179-250ul) at the dilution 1:400. Nuclei were counterstained with Hoechst dye.

For each condition, 25 or 30 pictures per well were automatically taken using Operetta^®^ (Perkin Elmer) with 20x magnification, using the same acquisition parameters. From images, analyses were directly and automatically performed by Harmony^®^ (Perkin Elmer). The following endpoints were automatically assessed: neuron survival (MAP-2 positive neurons, number of neurons), neurite network (MAP-2 staining, total neurite length, in μm), extranuclear TDP-43 in MAP-2 positive neurons (overlapping area between MAP-2 and extranuclear TDP-43, in μm^2^), LC3 in MAP-2 positive neurons (overlapping area between LC3 and MAP2+, in μm^2^), TDP-43(+) and LC3(+) co-localization (overlapping area between TDP-43 and LAMP2 within MAP-2 staining, in μm^2^).

#### Immunofluorescence analysis: neuronal survival, neurite network, LAMP2, and LC3

On culture day 13, test compounds were applied in vehicle (basal media, 0.1% DMSO, 0.05 ng/mL HGF) for 15 min. Without removal of test compound, glutamate (5 μM) was added to the culture for 20 min followed by wash-out and re-application of test compounds. Test compounds were refreshed every 24 h.

After 24 h in culture, cultures were fixed with 4% paraformaldehyde in PBS, pH 7.3 for 2 h at RT and then permeabilized with 0.1% saponin. Fixed permeabilized cultures were incubated with the following primary antibodies: chicken anti MAP2 (1:400) (Abcam; #Ab5392), rabbit anti LC3b (1:50) (Abcam; #Ab192890), and mouse anti lysosomal-associated membrane protein 2 (LAMP2) (1:1000) (Novus; #NB300-591). Primary antibodies were revealed with Alexa Fluor 488 anti-mouse IgG (Sigma-Aldrich; #SAB4600042-250ul) at the dilution 1:400, Alexa Fluor 568 anti-rabbit IgG (Sigma-Aldrich; #SAB4600084-250ul) at the dilution 1:400 and with CF™ 647 anti-chicken IgG (Sigma-Aldrich; #SAB4600179-250ul) at the dilution 1:400. Nuclei were counterstained with Hoechst dye.

For each condition, 25 or 30 pictures per well were automatically taken using Operetta^®^ (Perkin Elmer) with 20x magnification, using the same acquisition parameters. From images, analyses were directly and automatically performed by Harmony^®^ (Perkin Elmer). The following endpoints were automatically assessed: neuron survival (MAP-2 positive neurons, number of neurons), neurite network (MAP-2 staining, total neurite length, in μm), LAMP-2 in MAP-2 positive neurons (overlapping area between MAP-2 and LAMP2, in μm^2^), LC3 in MAP-2 positive neurons (overlapping area between LC3 and MAP2+, in μm^2^), LAMP2(+) and LC3(+) co-localization (overlapping area between LAMP2 and LC3 within MAP-2 staining, in μm^2^).

#### Western blot analysis: phospho-GSK3β and phospho-TDP-43

On culture day 13, test compounds were applied in vehicle (basal media, 0.1% DMSO, 0.05 ng/mL HGF) for 15 min. Without removal of test compound, glutamate (5 μM) was added to the culture for 20 min followed by wash-out and re-application of test compounds for 24 h. For biochemical endpoints, cells were lysed in CelLyticMT reagent (Sigma-Aldrich; #C3228-50ML) with 1% protease and phosphatase inhibitor cocktails in 60 μL per well. Protein quantification was performed by standard bicinchoninic acid (BCA) assay and target proteins were quantified by Western Blot automatic protein analysis via Simple Western (ProteinSimple) according to manufacturer’s instructions. Capillaries were incubated for 2 h with the following primary antibodies: anti GSK3β (Ozyme; #9315P), anti phospho-GSK3β (Ser9) (Ozyme; #9331S), anti TDP43 (Abcam; #ab104223), anti TDP43 (Ser 409/410) (Fisher; #17267423), and anti glyceraldehyde-3-phosphate dehydrogenase (GAPDH) (Ozyme; #2118S). After washing, capillaries were incubated with horseradish peroxide (HRP)-conjugated secondary antibodies (Bio-Techne; HAF005) for 1 h. Chemiluminescent substrate signal was quantified in the WES instrument and analysis performed in Compass (ProteinSimple) software. 4 biological replicates per condition were analyzed.

#### MET siRNA experiments

For siRNA experiments, the culture was transfected with the siRNA scrambled (control) or with the siRNA target (c-MET; entrezgene ID 24553) at 1 nM (validated to reduce MET expression by ~40%) (Supplementary Figure S6). The transfection was performed with the kit INTERFERin^®^ (Polyplus transfection). siRNA (smartpool grade) was purchased from Dharmacon. Following a 24 h siRNA transfection, glutamate toxicity assay was performed as described above to evaluate neuronal survival and neurite networks. HGF (5 ng/mL) was used as a positive control to demonstrate that activation of HGF signaling is neuroprotective, and that its neuroprotective activity is dependent on the MET receptor.

### MET activation assay

On culture day 13, motor neurons were treated with vehicle (basal media, 0.1% DMSO), HGF 0.05 ng/mL (subthreshold concentration of HGF) or HGF 0.05 ng/mL + ATH-1105 (100 nM, 500 nM, or 1 μM) for 15 min. Cells were lysed with a defined lysis buffer consisting of CelLyticMT reagent with 1% of Protease and phosphatase inhibitor cocktail (60 μL per well). For each condition, the quantity of protein was determined using the micro kit BCA (Pierce). Briefly, lysates were diluted at 1:20 in (PBS) and mixed in equal volume with a micro-BCA Working reagent. These solutions were incubated at 60 °C for 1 h and the quantity of protein was measured at 562 nm with a spectrophotometer Nanovue (GE Healthcare) and compared with the standard Bovin Serum Albumin curve (BSA, Pierce). All reagents (except primary and secondary antibodies) were provided by ProteinSimple^®^. They were prepared and used according to manufacturer’s recommendations for use on WES™ (ProteinSimple, United States). The levels of the following protein were assessed: anti-MET (Novus; #NBP2-44306), anti phospho-MET (ThermoFisher; #MA5-15083), and GAPDH (Ozyme; #2118S). Four biological replicates per condition were analyzed.

### Statistical analyses for *in vitro* assays

All statistical analyses were conducted with GraphPad Prism software (version 10; GraphPad Software, Inc., San Diego, CA). Data is represented as mean ± SEM. Statistical significance in in vitro assays was determined by one-way ANOVA followed by Fisher’s least significant difference. Comparisons were considered statistically significant at *p* < 0.05.

## Results

### Treatment with ATH-1105, riluzole, or their combination attenuates disease progression in the Prp-TDP43A315T mouse model of ALS

**Figure 1:**
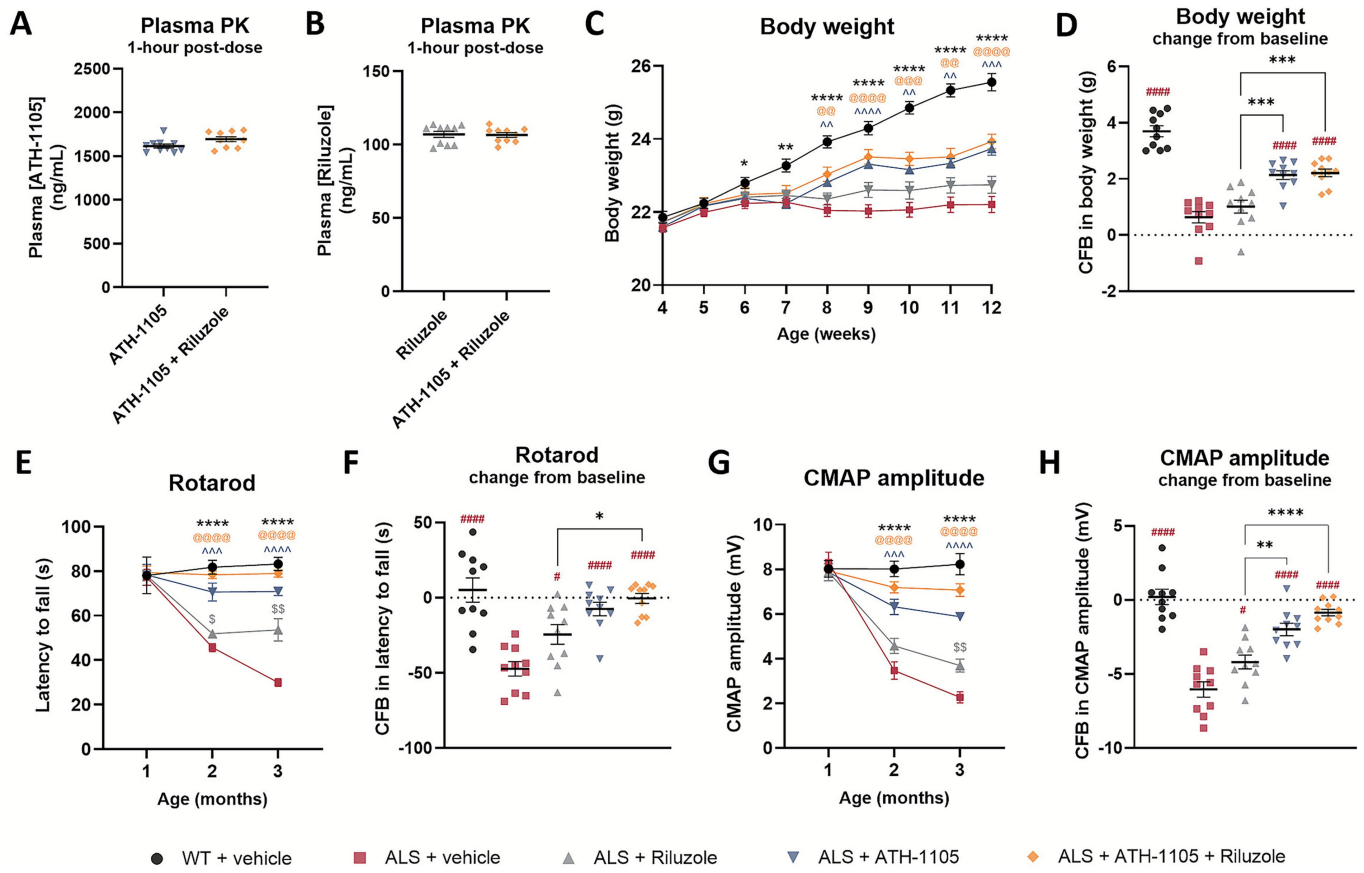
ATH-1105 and riluzole, alone or in combination, mitigate ALS-related neuromotor deficits in Prp-TDP43^A315T^ ALS mice. Plasma concentrations of **(A)** ATH-1105 and **(B)** riluzole from ALS mice measured one hour after dosing. Graphical representation of **(C)** body weight over time, in grams, and **(D)** body weight as a change from baseline (CFB; from 1 to 3 months of age). Motor function was measured via **(E)** rotarod performance over time and as a **(F)** CBF in latency to fall. Assessment of nerve function by **(G)** sciatic nerve compound muscle action potential (CMAP) amplitude over time and as a **(H)** CFB in CMAP. Data are presented as mean ± SEM; *n* = 10. Statistical differences in panels **(C,E,G)** determined via two-way ANOVA followed by Dunnett’s multiple comparisons test against the ALS + vehicle group. Symbols indicate comparisons to ALS + vehicle group as follows: “*” versus WT + vehicle, “$” versus ALS + Riluzole, “^” versus ALS + ATH-1105, and “@” versus ALS + ATH-1105 + Riluzole. For panels **(D,F,H)**, one-way ANOVA followed by Tukey’s multiple comparisons test was used, where “#” represents comparisons versus ALS + vehicle and “*” represents all other significant comparisons among ALS groups as indicated. The following applies to all symbols: ^*^ *p* < 0.05, ^**^ *p* < 0.01, ^***^ *p* < 0.001, ^****^ *p* < 0.0001.

To evaluate the efficacy of ATH-1105 or riluzole in a TDP-43-driven model of ALS, 1-month-old male Prp-TDP43^A315T^ transgenic mice (“ALS mice”) were treated with either ATH-1105 (20 mg/kg, p.o.), riluzole (5 mg/kg, i.p.), or the combination of the two compounds once daily for 2 months. Dose levels were selected based on previous studies by our group (16) and others (20–22). Quantification of acute plasma exposures did not suggest a pharmacokinetic interaction of the combined treatments on ATH-1105 plasma concentrations (Figure 1A) or on riluzole plasma concentrations (Figure 1B) compared to their respective treatments alone.

Body weights, measured weekly, showed an absence of normal weight gain over time in ALS mice treated with vehicle, an effect which reflects an important clinical feature of ALS (23). In comparison, body weight gain across the study duration was significantly improved by treatment with ATH-1105 or the combination of ATH-1105 and riluzole, but not by riluzole when administered alone (Figure 1C). Indeed, calculating the change in body weight from baseline across groups revealed a significantly greater benefit of ATH-1105 or combination treatment when directly compared to riluzole (Figure 1D).

Progressive loss of motor and nerve function are fundamental aspects of ALS; these deficits develop gradually starting from 1 month of age in ALS mice. Motor function, assessed via latency to fall in the rotarod task, declined progressively in ALS mice treated with vehicle. This decline was mitigated by daily treatment with either riluzole, ATH-1105, or their combination (Figure 1E). Interestingly, when plotted as a change in rotarod performance from the 1-month baseline, mice receiving both ATH-1105 and riluzole exhibited no decline in motor function over 2 months compared to WT animals, whereas the vehicle-treated ALS animals experienced a deterioration in their performance of nearly 50 s within the same period (Figure 1F). Similar improvements were observed in other motor function tests, namely balance beam (Supplementary Figures S1A,B), grip test (Supplementary Figures S1C,D), and Kondziela inverted screen test (Supplementary Figures S1E,F).

ALS mice treated with vehicle also exhibited a marked decrease in sciatic nerve compound muscle action potential (CMAP) amplitude, an electrophysiological measure of neuromuscular connectivity and function, from 1 to 3 months of age (Figure 1G). Daily treatment with riluzole partially mitigated this decline, with significant effects observed only at 3 months. Treatment with ATH-1105 alone significantly reduced nerve dysfunction at both 2 and 3 months, as did the combination of ATH-1105 and riluzole. A change from baseline analysis indicated that ATH-1105, alone or in combination with riluzole, was more effective than riluzole alone, with the combination treatment group having the best protection against nerve-related dysfunction (Figure 1H). Nerve conduction velocity (NCV), another clinically relevant electrophysiological measure related to axon myelination, displayed similar patterns of deterioration in ALS mice with one exception: the riluzole group did not demonstrate significant mitigation of the overall change from baseline in NCV (Supplementary Figures S1G,H). Together, these results demonstrate that both ATH-1105 and riluzole had significant efficacy in the Prp-TDP43^A315T^ mouse model of ALS, with ATH-1105 showing a larger effect size under the conditions tested. Furthermore, the combination of ATH-1105 and riluzole proved more effective than riluzole alone. Although the combination was not statistically better than ATH-1105 alone by these measures, the combination group exhibited the best effect size of all treatment groups tested, often nearing no decline in function over 2 months in this transgenic model of ALS.

### ATH-1105, but not riluzole, preserves large-diameter axons and myelination in sciatic nerves of ALS mice

**Figure 2:**
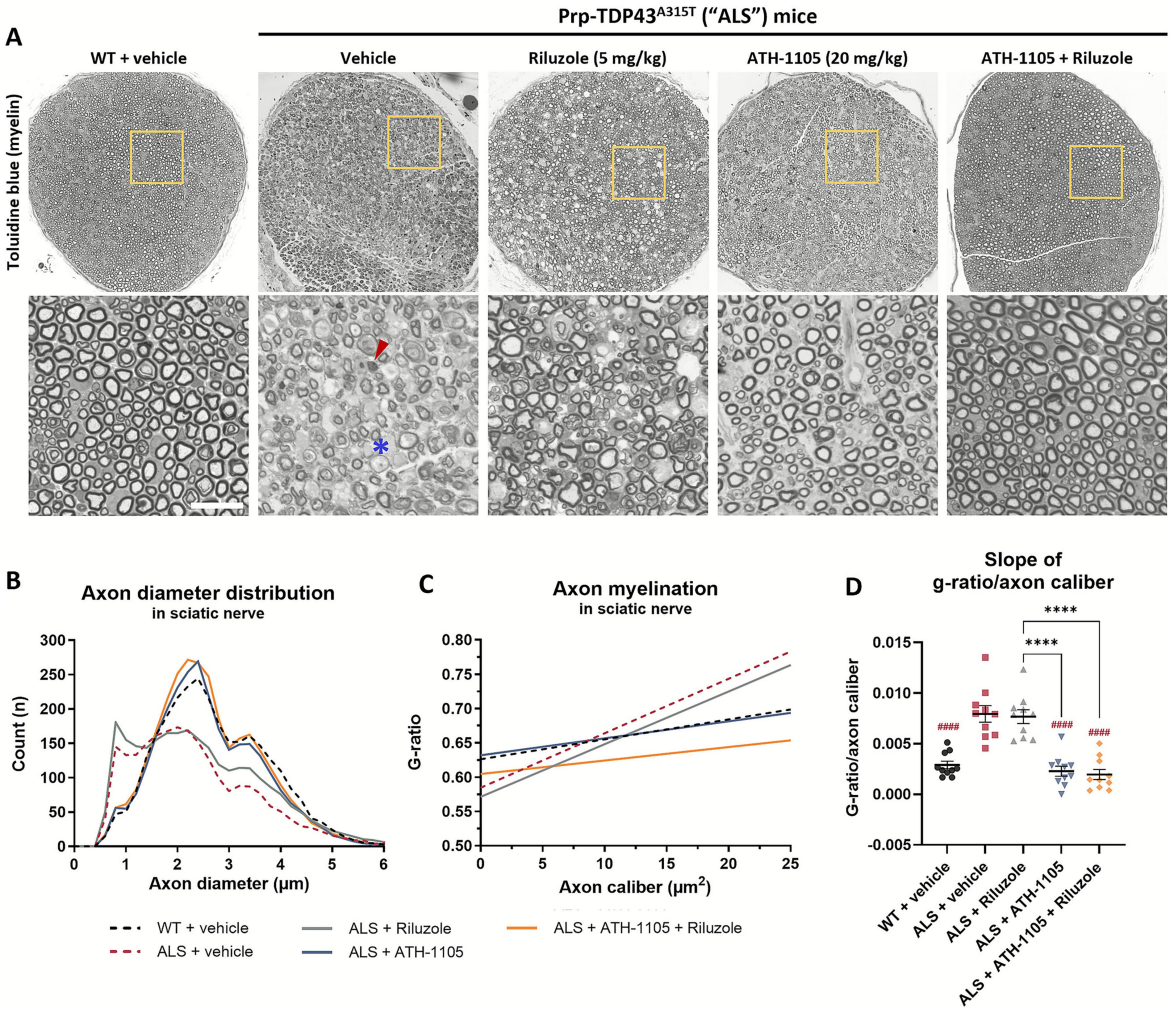
ATH-1105 significantly preserves normal axonal distributions and myelination in ALS mice while treatment with riluzole does not. **(A)** Representative images of sciatic nerve semithin cross sections from 3-month-old WT or ALS mice stained with toluidine blue for myelin, following 2 months of respective treatments. Scale bar = 20 μm. Red arrow indicates an example of degenerating axons. Blue asterisk indicates an example of demyelinated axons. **(B)** Quantification of axonal diameters, in μm, as a frequency distribution per group. Graphical representation of axon myelination via **(C)** regression lines derived from analysis of g-ratio versus axon caliber (μm^2^) per group, and **(D)** corresponding slopes from g-ratio/axon caliber regression lines. Data in **(D)** are presented as mean ± SEM; *n* = 10. Statistical differences were determined by one-way ANOVA followed by Tukey’s multiple comparisons test. ^####^ *p* < 0.0001 versus ALS + vehicle. ^****^ *p* < 0.0001 versus indicated groups.

Sciatic nerves collected at 3 months of age were sectioned and stained with toluidine blue to visualize myelin and axons (Figure 2A). Quantification of axon diameter distributions revealed a selective loss of larger-diameter axons in ALS mice treated with vehicle (Figure 2B), in line with the motor neuron dysfunction observed in this model. An additional population of small diameter axons was noted in ALS mice, which is likely the result of large-diameter axons shrinking in the process of degeneration (24). The sciatic nerves from ALS mice treated with riluzole displayed a similarly abnormal distribution of axons, with only a slight preservation of axon numbers, most notable in the population ≥ 3 μm in diameter. On the other hand, ALS mice treated with ATH-1105 alone or in combination with riluzole showed axon diameter distributions similar to WT sciatic nerves.

Myelination, another important aspect of proper nerve signal transduction, was measured by calculating the g-ratio: the area of the inner nerve fiber diameter (axon only) over the outer diameter (axon + myelin) (25). A greater g-ratio indicates thinner myelin, and the theoretical optimal score for peripheral nerve conduction near 0.6 (25). To consider myelination as a function of axon size, g-ratios were plotted against axon area (caliber), in μm^2^. In WT animals, we observed a relatively consistent degree of myelination across all calibers of myelinated axons (Figure 2C). In ALS animals, we found an abnormal pattern of g-ratio distribution, resulting in steeper average regression lines than in the WT group (Figure 2D) driven by a population of larger caliber axons (~ ≥ 7.5 μm^2^) with higher g-ratios (≥0.7) (Supplementary Figure S3A). This indicates demyelination of these large axons, in agreement with the reduced NCV previously reported (Supplementary Figures S1G,H). Additionally, a higher density of small-caliber axons (<2.5 μm^2^) with relatively low g-ratios (≤0.5) can be observed, indicating that these are likely residual axonal structures surrounded by a compact myelin layer, again consistent with a neurodegenerative process. Treatment with riluzole in ALS mice did not overcome the ALS-related deficits in myelination, with ALS mice treated with riluzole exhibiting similar g-ratio distributions and overall regression slopes as those treated with vehicle only (Supplementary Figure S3B). Treatment with ATH-1105 (Supplementary Figure S3C) or ATH-1105 and riluzole in combination (Supplementary Figure S3D) had a significant protective effect on axon myelination, with the resulting distribution of g-ratios across axon calibers, as well as average slopes calculated from these regression lines, being similar to WT. Overall, the histopathological analysis of sciatic nerves corroborates the treatment effects observed using in-life electrophysiological measures. It further supports the distinctly potent neuroprotective effect of ATH-1105 compared to riluzole in a model of ALS- an effect maintained when the two compounds are administered in combination.

### Differential effects of ATH-1105 compared to riluzole on ALS-related biomarkers of neurodegeneration, inflammation, and TDP-43 protein pathology

**Figure 3:**
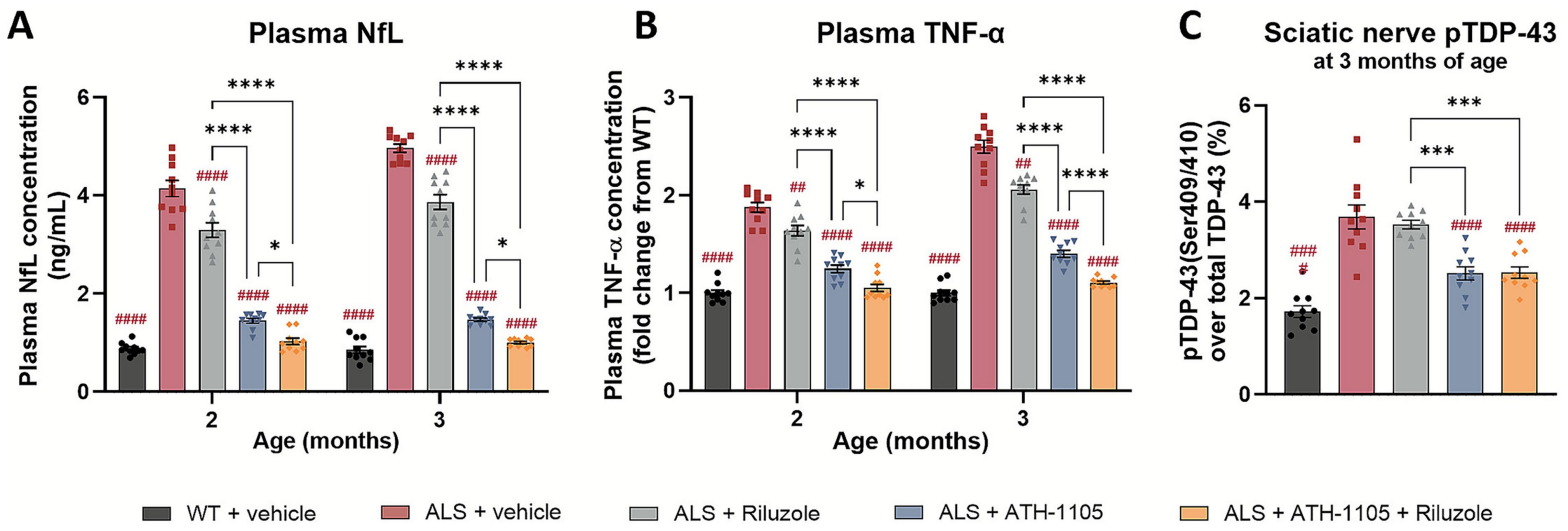
ATH-1105 demonstrates greater attenuation of disease-relevant biomarkers compared to riluzole in ALS mice. Plasma concentrations of **(A)** NfL, in ng/mL, and **(B)** TNF-*α*, as a fold change from WT concentration, after 1 and 2 months of each respective treatment (at 2 and 3 months of age). **(C)** TDP-43 phosphorylated at Ser409/410 as a percent of total TDP-43 measured in sciatic nerve homogenate at 3 months of age, following 2 months of respective treatment. Data are presented as mean ± SEM; *n* = 10. Statistical differences were determined by **(A,B)** two-way ANOVA or **(C)** one-way ANOVA followed by Tukey’s multiple comparisons test. “#” represents comparisons versus ALS + vehicle, “*” represents all other significant comparisons among ALS groups as indicated. The following applies to all symbols: ^*^ *p* < 0.05, ^**^ *p* < 0.01, ^***^ *p* < 0.001, ^****^ *p* < 0.0001.

The Prp-TDP43^A315T^ mouse model of ALS also recapitulates changes in clinically relevant plasma biomarkers associated with disease progression. NfL, a well-established fluid biomarker in clinical ALS, is a key axonal protein that correlates strongly with ongoing neurodegeneration when measured in plasma or cerebrospinal fluid (CSF) (26, 27). As such, NfL is emerging as an important potential surrogate biomarker in clinical trials testing novel therapeutics aimed at slowing neurodegeneration (28). In our study, ALS mice showed a significant elevation in plasma NfL at both 2 and 3 months of age (Figure 3A), indicating high rates of neurodegeneration in-line with the pathophysiology of ALS. Riluzole treatment modestly, but significantly, reduced plasma NfL levels at both time points, a notable finding given the limited research on riluzole’s effects on NfL in preclinical or clinical ALS settings (29). Treatment with ATH-1105 strongly reduced plasma NfL levels at both 2 and 3 months of age, and the magnitude of this change was significantly greater than that of riluzole. The combination treatment of ATH-1105 and riluzole further reduced plasma NfL, to a degree that statistically outperformed either treatment alone. Notably, the NfL levels in mice given the combination of treatments were comparable to levels in WT mice. The magnitude of each treatment’s effects on NfL levels closely aligned with improvements in motor and nerve function readouts in our study, validating the relationship between NfL/neurodegeneration and the degree of neuromuscular dysfunction in this mouse model of ALS.

Levels of pro-inflammatory cytokines, including TNF-*α*, are clinically relevant biomarkers of systemic inflammation in ALS (30). Similarly, in ALS mice treated with vehicle, TNF-α levels are increased compared to WT animals (Figure 3B). Daily treatment with riluzole, ATH-1105, or their combination significantly reduced this inflammatory response. As with plasma NfL levels, treatment with ATH-1105 significantly outperformed riluzole at the doses tested, and the combination of both treatments further reduced TNF-α levels compared to each treatment alone. A similar pattern was observed with plasma IL-6, another pro-inflammatory marker relevant to clinical ALS (Supplementary Figure S2A).

The final disease-related biomarker assessed was pTDP-43. ALS mice treated with vehicle exhibited significantly elevated levels of TDP-43, phosphorylated at Ser409/410, compared to their WT counterparts (Figure 3C) as measured using ELISA in sciatic nerves homogenates collected from mice at 3 months of age. Increased phosphorylation at this site has also been observed in ALS patients (31). We found that, although riluzole treatment led to significant improvements in motor function, nerve function, and other plasma biomarkers in this model of ALS, it did not impact levels of pTDP-43. In contrast, ATH-1105, alone or in combination with riluzole, significantly reduced pTDP-43 levels. This suggests that ATH-1105 may have additional disease-modifying potential not observed with riluzole.

### ATH-1105 enhances neuronal survival and neurite network, mitigates extranuclear TDP-43 accumulation, and reduces markers of autophagic stress in primary motor neuron culture

**Figure 4:**
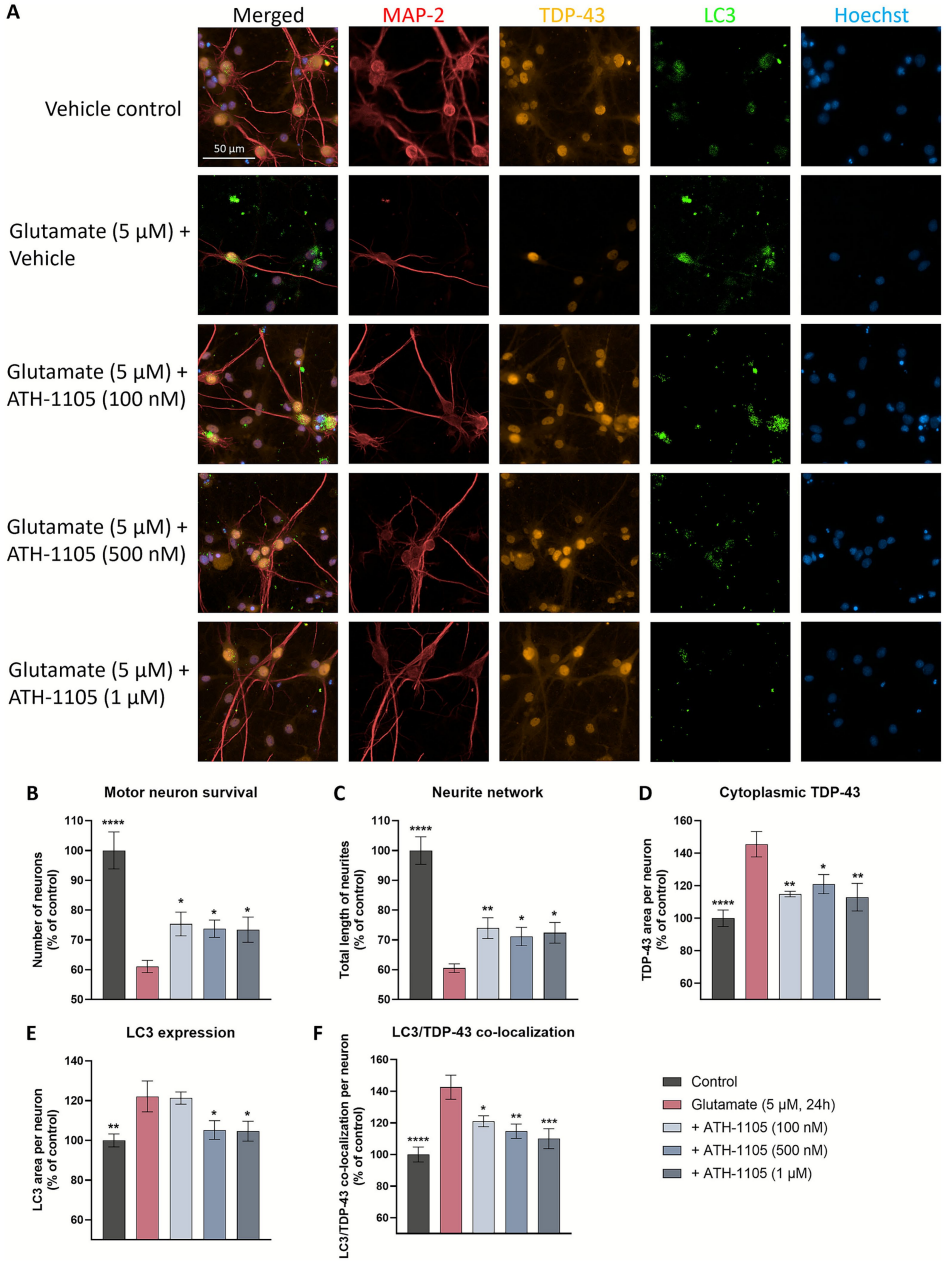
ATH-1105 enhances neuronal survival and neurite network integrity, and reduces cytoplasmic accumulation of extranuclear TDP-43, LC3 expression, and co-localization of TDP-43 and LC3 in glutamate-challenged motor neuron cultures. **(A)** Primary rat spinal motor neurons were treated with ATH-1105 and glutamate (5 μM) for 24 h and immunolabeled with microtubule-associated protein-2 (MAP2; neuronal marker), anti-TDP-43, and anti-LC3. Scale bar = 50 μm. Quantification of **(B)** motor neuron survival (number of MAP2 + neurons), **(C)** neurite network (total length of MAP2 + neurites), **(D)** cytoplasmic TDP-43 levels within MAP2 + staining, **(E)** LC3 + autophagosomes within MAP2 + staining, and **(F)** LC3/TDP-43 co-localization within MAP2 + staining, expressed as area per neuron. Data are expressed as percentage of normal control (100%) and presented as mean ± SEM; *n* = 4–6 (1 biological replicate). Statistical differences were determined by one-way ANOVA followed by Fisher’s LSD test versus glutamate control. ^*^ *p* < 0.05, ^**^ *p* < 0.01, ^***^ *p* < 0.001, ^****^ *p* < 0.0001.

**Figure 5:**
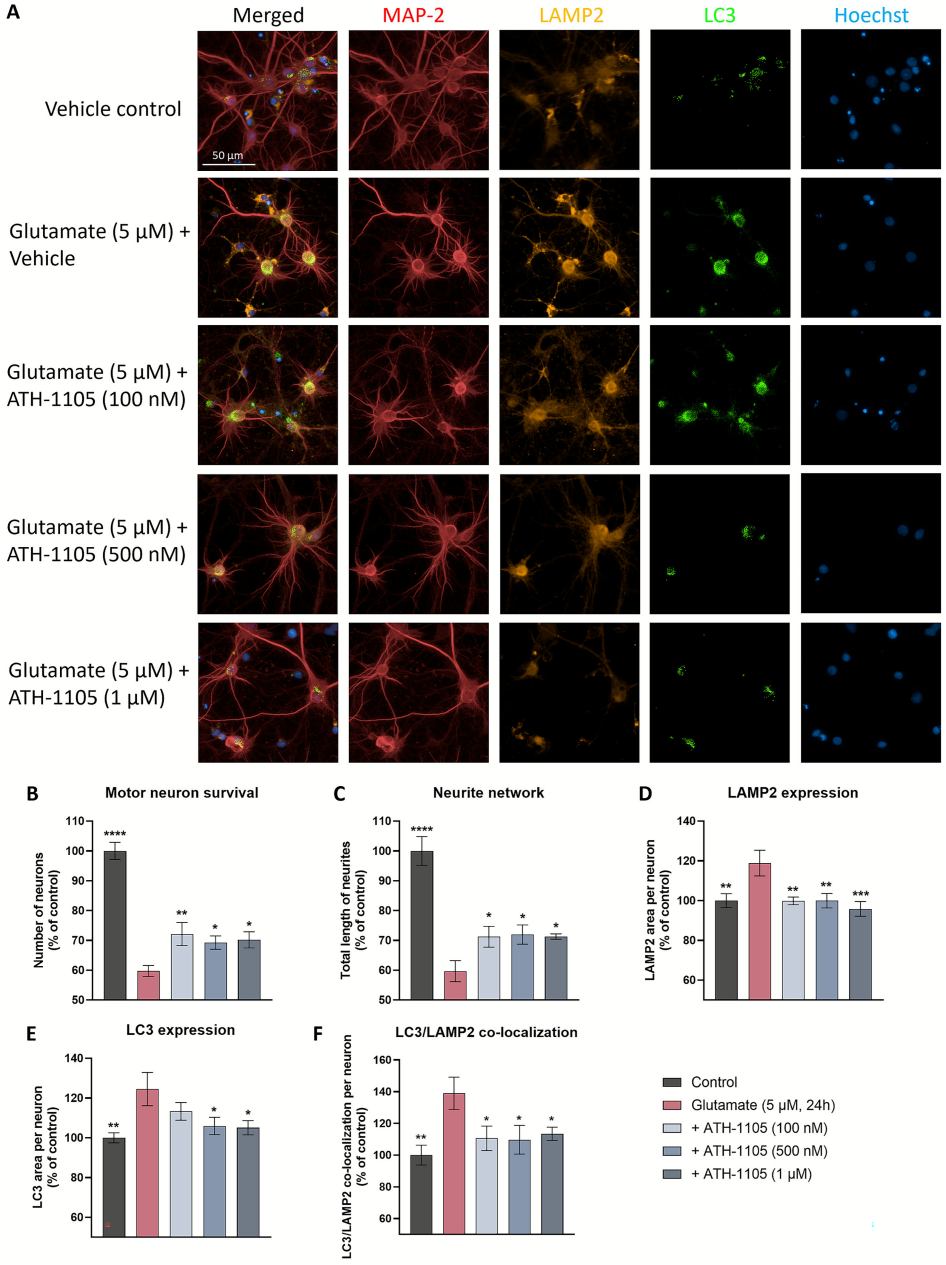
ATH-1105 enhances neuronal survival and neurite network integrity, and reduces LAMP2 expression, LC3 expression, and co-localization of LAMP2 and LC3 in glutamate-challenged motor neuron cultures. **(A)** Primary rat spinal motor neurons were treated with ATH-1105 and glutamate (5 μM) for 24 h and immunolabeled with microtubule-associated protein-2 (MAP2; neuronal marker), anti-LAMP2, and anti-LC3. Scale bar = 50 μm. Quantification of **(B)** motor neuron survival (number of MAP2 + neurons), **(C)** neurite network (total length of MAP2 + neurites), **(D)** LAMP2 + lysosomes within MAP2 + staining, **(E)** LC3 + autophagosomes within MAP2 + staining, and **(F)** LC3/LAMP2 co-localization within MAP2 + staining, expressed as area per neuron. Data are expressed as percentage of normal control (100%) and presented as mean ± SEM; *n* = 4–6 (1 biological replicate). Statistical differences were determined by one-way ANOVA followed by Fisher’s LSD test versus glutamate control. ^*^ *p* < 0.05, ^**^ *p* < 0.01, ^***^ *p* < 0.001, ^****^ *p* < 0.0001.

Mitigating TDP-43 protein dysfunction is the aim of a number of novel therapeutic targets in development for ALS, i.e., STMN2 (32), UNC13A (33), eIF2B (34), PIKFYVE (35). As such, we sought to further characterize the impact of ATH-1105 on TDP-43 pathology following glutamatergic stress, a key aspect of ALS pathogenesis. Following a 24 h glutamate challenge in primary rat spinal motor neuron cultures, co-immunostaining of MAP2, TDP-43, and LC3 was conducted to evaluate motor neuron survival, total length of neurites, extranuclear TDP-43, LC3 expression, and LC3/TDP-43 co-localization (Figure 4A). We found that after 24 h of glutamate exposure, spinal motor neuron survival (Figure 4B) and total length of neurites (i.e., neurite network) (Figure 4C) significantly decreased. Treatment with ATH-1105 (100 nM, 500 nM, or 1 uM) counteracted these effects; that is, ATH-1105 treatment led to a significant increase in neuronal survival (Figure 4B) and neurite network integrity (Figure 4C) relative to glutamate control. Furthermore, excitotoxic injury was found to significantly increase extranuclear TDP-43 levels, while treatment with ATH-1105 (100 nM, 500 nM, or 1 μM) significantly reduced this pathological mis-localization at all concentrations tested (Figure 4D). To better understand the effect of ATH-1105 on extranuclear TDP-43 associated with glutamate toxicity, we assessed the impact of ATH-1105 on static markers of autophagy, which is an essential cellular process that mediates the degradation of pathological protein aggregates such as TDP-43 and has been shown to be disrupted in ALS (6, 7). Disruption of autophagic function may manifest as the abnormal accumulation of autophagic proteins. Indeed, we found that levels of LC3b (autophagosome marker) and co-localization of LC3 and TDP-43 were elevated following the 24 h excitotoxic challenge. Interestingly, treatment with ATH-1105 at 500 nM and 1 μM significantly reduced levels of LC3 (Figure 4E) and co-localization of LC3 with TDP-43 (Figure 4F), potentially reflecting an alleviation of autophagic stress. To gain additional insight into the effect of ATH-1105 on autophagic pathway function, we also assessed the impact of ATH-1105 on LC3 expression in conjunction with the expression of lysosome-associated membrane protein 2 (LAMP2), a protein that is dysregulated in association with lysosomal stress (Figure 5A). Here again, we found that ATH-1105 enhanced motor neuron survival (Figure 5B) and neurite network integrity (Figure 5C) following toxic glutamate exposure. In addition, we found that LAMP2 and LC3 levels were increased in glutamate-challenged cultures, an effect which was reduced with ATH-1105 treatment (Figures 5D,E). Furthermore, we also noted a significant increase in LC3/LAMP2 co-localization after the 24 h glutamate challenge (Figure 5F), which may reflect a blockade of autophagic flux. Here again, treatment with ATH-1105 significantly attenuated the increase of LC3/LAMP2 co-localization. Taken together, these observations suggest that ATH-1105 attenuates glutamate-mediated autophagic stress, which may play a role in the observed reductions of extranuclear TDP-43.

### ATH-1105 reduces TDP-43 phosphorylation at Ser409/410 and increases GSK3β phosphorylation at Ser9

**Figure 6:**
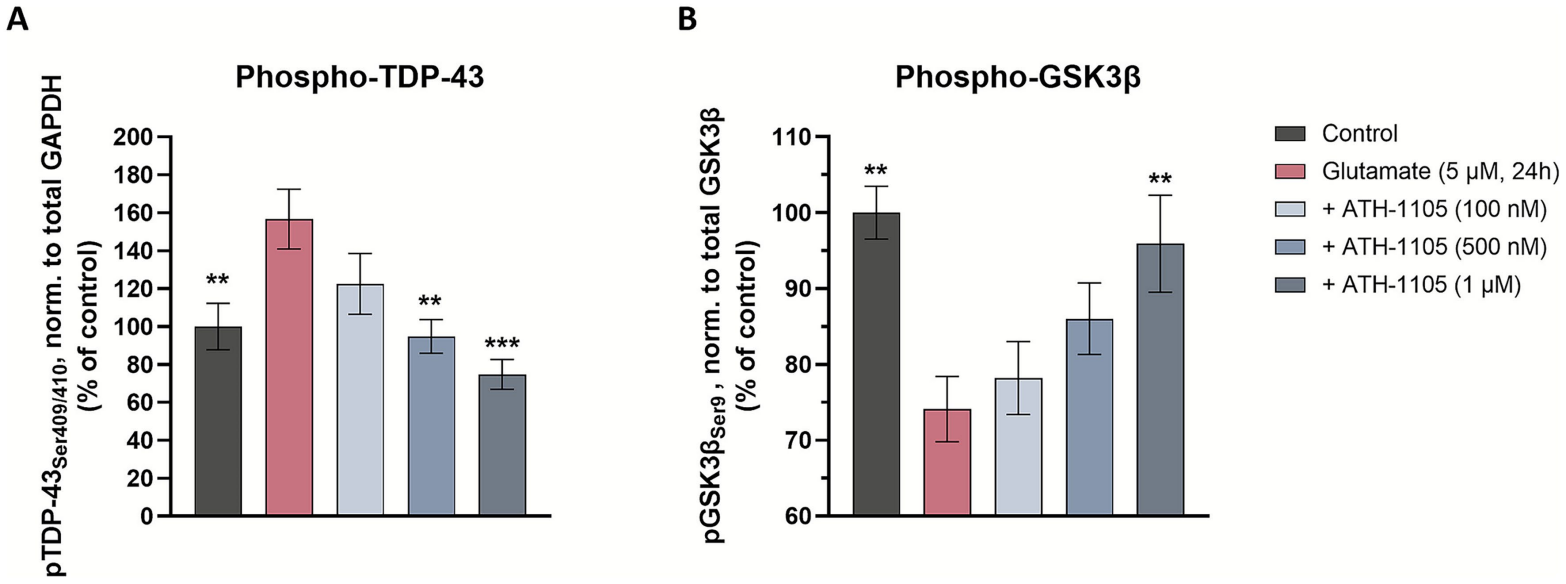
ATH-1105 reduces TDP-43 phosphorylation at Ser409/410 and increases GSK3β phosphorylation at Ser9. Primary rat spinal motor neurons were treated with ATH-1105 and glutamate (5 μM) for 24 h and cell culture lysates were analyzed for phospho-TDP-43 (pTDP-43^Ser409/410^), GAPDH, phospho-GSK3β (pGSK3β^Ser9^), and total GSK3β via automated western blotting (Simple Western). Quantification of **(A)** pTDP-43 (Ser409/410), normalized to GAPDH level and **(B)** pGSK3β (Ser9), normalized to total GSK3β level in cell culture lysates. Data are expressed as percentage of normal control (100%) and presented as mean ± SEM; *n* = 3–4 biological replicates. Statistical differences were determined by one-way ANOVA followed by Fisher’s LSD test versus glutamate control. ^*^ *p* < 0.05, ^**^ *p* < 0.01, ^***^ *p* < 0.001, ^****^ *p* < 0.0001.

In addition to autophagic disruption, there are other cellular processes that can exacerbate ALS-related TDP-43 pathology. Phosphorylation of extranuclear TDP-43 is known to contribute to the toxic accumulation of this protein (4, 36). Indeed, phosphorylation of TDP-43 at the Ser409/410 site is a hallmark of clinical ALS pathology in impacted nervous system tissues, as well as in preclinical ALS models (including the Prp-TDP43^A315T^ mouse model, as reported above in Figure 3C) (4). To assess the effect of ATH-1105 on pTDP-43 (Ser409/410) *in vitro*, spinal motor neurons were treated with ATH-1105 and excitotoxic glutamate, and western blotting (via automated Simple Western) was conducted to assess pTDP-43 (Supplementary Figure S4A). Quantification of Simple Western showed that glutamate exposure resulted in a significant increase in pTDP-43(Ser409/410) compared to the unchallenged control (Figure 6A). In agreement with our *in vivo* findings, ATH-1105 treatment again significantly reduced levels of pTDP-43, reaching statistical significance at the 500 nM and 1 μM dose levels.

One factor known to be modulated by the HGF pathway is glycogen synthase kinase 3β (GSK3β) (37), a major kinase that is known to promote the phosphorylation of pathological proteins such as TDP-43 (38, 39). Thus, we hypothesized that ATH-1105 may impart an effect on GSK3β activity. To test this, western blotting (via automated Simple Western) was utilized to evaluate the effect of ATH-1105 on GSK3β phosphorylation at Ser9 (Supplementary Figure S4B), a phosphorylation site that is known to inhibit GSK3β activity. In spinal motor neurons, we found that application of glutamate induced a significant decrease in the ratio of pGSK3β/GSK3β (Figure 6B), indicating an increase in GSK3β activity, which may promote the phosphorylation of its target proteins, including TDP-43. Treatment with ATH-1105 increased pGSK3β/GSK3β levels at the highest concentration of 1 μM, suggesting a decrease in GSK3β activation, an effect that may be involved in reduction of TDP-43 phosphorylation in motor neurons.

### The neuroprotective effects of ATH-1105 on motor neurons are dependent on HGF/MET system signaling

Next, we asked if ATH-1105 was exerting its effects via the canonical HGF receptor, MET. A series of experiments were designed to verify that the neuroprotective actions of ATH-1105 are mediated through HGF signaling via MET receptor activation. Namely, we tested the ability of ATH-1105 to positively modulate HGF-mediated MET receptor activation in a primary culture of rat spinal motor neurons. Cultures were exposed to a low level of exogenous HGF (0.05 ng/mL) for 15 min, either alone or in the presence of ATH-1105 (100 nM, 500 nM, or 1 μM). MET receptor activation was assessed by measuring phosphorylation at Tyr1234/1235. Results showed that while subthreshold level of HGF did not itself induce a significant increase in MET phosphorylation, the addition of ATH-1105 at all tested levels resulted in significant elevations in MET phosphorylation compared to untreated controls (Supplementary Figures S5A,B).

**Figure 7:**
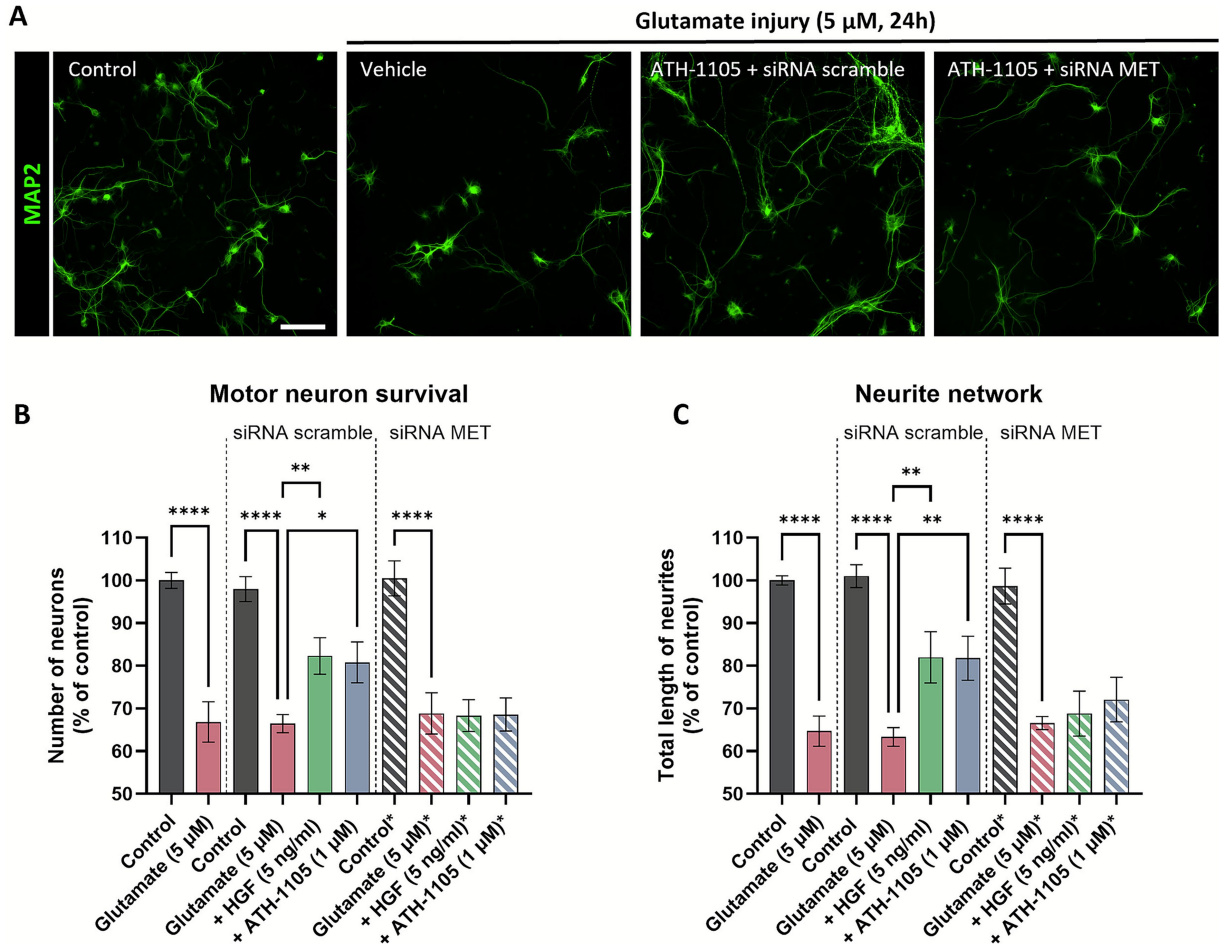
The neuroprotective effects of ATH-1105 in spinal motor neurons are mediated via the MET receptor. **(A)** Representative images of primary rat spinal motor neurons labeled with MAP-2, showing the effect of a 24 h 5 μM glutamate challenge ± ATH-1105 (1 μM) in the presence of either scramble or MET-targeted siRNA. Scale bar = 100 μm. Quantification of **(B)** motor neuron survival (number of MAP2 + neurons) and **(C)** neurite network integrity (total length of MAP2 + neurites) following treatment with 5 ng/mL HGF or 1 μM ATH-1105, in the presence of either scramble or MET-targeted siRNA. Data are expressed as percentage of normal control and presented as mean ± SEM; *n* = 4–6 (1 biological replicate). Statistical differences were determined by one-way ANOVA followed by Fisher’s LSD test versus glutamate control. ^*^ *p* < 0.05, ^**^ *p* < 0.01, ^***^ *p* < 0.001, ^****^ *p* < 0.0001.

To test whether MET receptor activation is required for the neuroprotective effects of ATH-1105 in motor neurons, we employed a small interfering RNA (siRNA) approach. An siRNA targeting the MET receptor was developed and validated in rat primary spinal motor neuron culture. Following siRNA transfection, the MET siRNA administered at 1 nM concentrations was found to decrease expression of both MET protein and mRNA by ~30–40%, while having no effect on number of neurons or cells after 24 h (Supplementary Figure S6). In the experimental phase, the neuroprotective effects of ATH-1105 (1 μM) against glutamate toxicity were assessed in the presence of the MET siRNA or a scramble siRNA control, using MAP2 immunostaining to evaluate motor neuron survival and neurite network integrity (Figure 7A). HGF treatment at 5 ng/mL was used as a positive control. Here again, exposure to glutamate resulted in a significant reduction in motor neuron numbers (Figure 7B) and total length of neurites (Figure 7C). In the presence of the scramble siRNA, both HGF and ATH-1105 treatment resulted in significant protection against these pathological changes. In the presence of the MET siRNA, however, the effects of both HGF and ATH-1105 were abolished. These findings confirm that the neuroprotective effects of ATH-1105 are mediated through the MET receptor.

## Discussion

The work presented builds on our findings that ATH-1105 consistently and robustly attenuates disease progression in the Prp-TDP43^A315T^ mouse model of ALS. It demonstrates that ATH-1105 substantially improves neuromuscular function and protects against body weight loss, while reducing markers of neurodegeneration, inflammation, and TDP-43 protein pathology in this model. We now show that the therapeutic benefits of ATH-1105 may be complementary to those of riluzole in this preclinical context. By several disease metrics, including motor and nerve function and biomarkers of inflammation and neurodegeneration, the combination of ATH-1105 and riluzole had a greater effect than either treatment alone. Testing novel therapeutics alongside riluzole is an important consideration in ALS drug development where restrictions on the use of standard of care therapies for the purposes of a clinical trial may be considered unethical. Importantly, our studies presented here also report a dose and route of riluzole that shows efficacy against several key components of ALS in a TDP-43-based preclinical model. This is noteworthy given that riluzole originally demonstrated preclinical efficacy in the SOD1^G93A^ mouse model of ALS (40), and more recent studies have failed to replicate these findings or extend them to other transgenic models of ALS (21, 41, 42). Our results showing a modest effect of riluzole on several endpoints in the Prp-TDP43^A315T^ mouse model of ALS are consistent with the reported clinical benefits of riluzole (11). It is therefore notable that within this context, ATH-1105 showed a greater degree of efficacy than riluzole.

Although both ATH-1105 and riluzole demonstrated efficacy in our studies, there were some critical differences in addition to the overall magnitude of their effects. Riluzole, as an anti-glutamatergic agent, largely slows disease progression by protecting against excitotoxicity-driven neuronal damage and degeneration (43). We found that while riluzole did have modest neuroprotective effects and offered partial protection against motor and nerve dysfunction *in vivo*, it did not address axon degeneration, demyelination, or pTDP-43 accumulation in the sciatic nerve. This supports the notion that targeting additional aspects of ALS, along with excitotoxicity, may improve the prognosis of ALS. ATH-1105, as a positive modulator of neurotrophic HGF signaling, is expected to have pleiotropic activity across multiple cell types involved in ALS, including neurons, astrocytes, microglia, macrophages, and muscle cells (16). Given the high expression of HGF/MET in Schwann cells (44), it may be the case that ATH-1105 also has a direct effect on these myelinating cell types as well, although this requires further investigation. We have also previously demonstrated that ATH-1105 is protective against multiple insults beyond glutamate excitotoxicity, including oxidative stress, mitochondrial dysfunction, and neuroinflammation (16). Here, we present for the first time that the neuroprotective effects of ATH-1105 in motor neurons are directly mediated via MET receptor activity using a MET siRNA approach, thereby validating its mechanism of action in a relevant cell type. Via these multimodal actions through the MET receptor, ATH-1105 may offer a comprehensive therapeutic strategy by targeting multiple pathological processes across a range of affected cell types in ALS. Future studies will aim to demonstrate target engagement of HGF/MET signaling and treatment effects in induced pluripotent stem cell-derived motor neurons from ALS patients as a surrogate for activity in human tissues.

The unique impact of ATH-1105 on TDP-43 protein pathology in vivo, which was not observed with riluzole under the same conditions, led us to investigate these effects more closely *in vitro*. In cultured primary neurons, ATH-1105 effectively reduces aberrant extranuclear TDP-43 and pTDP-43 accumulation, and led to a reduction of static markers of autophagic stress and an increase in GSK3β phosphorylation. In the context of ALS, autophagy is a crucial process for not only degrading and recycling damaged cellular components, but managing the pathological accumulation of proteins, notably TDP-43 (8). Under physiological conditions in healthy tissues, TDP-43 stabilizes mRNA levels of proteins involved in the autophagic process, including LC3, which is central in cargo selection and autophagosome biogenesis (45, 46). Elevated levels of extranuclear TDP-43 and LC3 as well as abnormal accumulation of autophagic vesicles have been observed in the spinal cord of people with ALS, with increased LC3 also being detectable in skin biopsies (47–49). In addition, we observed a significant increase in the expression of LAMP2 as well as increased co-localization of LC3 and LAMP2 following glutamate application, signifying increased lysosomal stress and autophagic impairment. The ability of ATH-1105 to mitigate abnormal accumulation of autophagic stress markers associated with glutamate toxicity may therefore suggests the compound may impart an impact on autophagic function, enabling the cell to better manage the degradation of these protein aggregates. However, additional experiments that directly assess the dynamic behavior of autophagy-related structures (e.g., LC3 turnover) and/or evaluation of lysosomal-associated machinery are required to fully characterize this effect.

Another mechanistic avenue explored in our studies was the effect of ATH-1105 on a key player in TDP-43 phosphorylation, GSK3β. This kinase is involved in numerous cellular processes, and its dysregulation has been associated with neurodegenerative disorders including ALS, where it has been found to be elevated in spinal cord and brain tissues (50–52). Recent studies have suggested that pathogenic TDP-43 is associated with aberrant activation of GSK3β in several models (39, 53, 54) including ALS patient-derived models. The activity of GSK3β has been shown to be tightly regulated via phosphorylation; for example, phosphorylation of GSK3β at Ser9 has been shown to inhibit its activity (55). In our motor neuron culture system, where the excitotoxic glutamate challenge was used to mimic an ALS-like environment, we observed a shift towards GSK3β activation (via reduced phosphorylation at Ser9), an effect that occurred in conjunction with an increase in pTDP-43. The ability of ATH-1105 to reduce GSK3β activity (via increased phosphorylation at Ser9) and pTDP-43 suggests that it may interrupt this pathological interplay. These actions of ATH-1105, may in part, be traced downstream of MET receptor activation, which may lead to phospho-inactivation of GSK3β via the PI3K/AKT pathway (52, 56).

### Limitations and future directions

Our *in vivo* studies were conducted in male mice as males develop a more aggressive ALS phenotype than female mice (18). While this allowed us to evaluate the animal population with the most severe progressive phenotype, it may also limit the generalizability of our findings. Additionally, while our in vivo analyses of peripheral nerve, neuromuscular function, and plasma biomarkers have strong translational relevance, they do not capture the full extent of CNS involvement in ALS. Future studies may aim to incorporate additional histopathological and biochemical analyses of the brain and spinal cord, including those related to upper and lower motor neuron survival. Similarly, although we observed significant reductions in pTDP-43 in the sciatic nerve, we did not assess its status in the CNS. Future studies can focus on determining whether ATH-1105 restores nuclear TDP-43 localization and function in the spinal cord and motor cortex, including its regulation of TDP-43-dependent transcripts such as STMN2 and UNC13A. This will allow for a more comprehensive understanding of the effect of ATH-1105 on both gain- and loss-of-function aspects of TDP-43 pathology.

*In vitro*, our interpretation of autophagic modulation by ATH-1105 is based on static markers (LC3, LAMP2, and co-localization with TDP-43) rather than direct assessments of autophagic flux. While the observed changes suggest normalization of autophagic proteins, future studies employing dynamic assays (e.g., LC3 turnover in the presence of bafilomycin A1) and lysosomal activity measurements are necessary to more precisely characterize ATH-1105’s effect on protein clearance pathways. In addition, while our data suggest that ATH-1105 reduces TDP-43 phosphorylation and this in part may be due to inhibition of GSK3β, additional work is required to establish a more direct link. Furthermore, future *in vitro* studies may also look to incorporate riluzole and combination treatments to assess potential mechanistic synergy at the cellular level.

## Conclusion

In summary, the work outlined above supports the therapeutic potential of ATH-1105 for the treatment of ALS, either as a monotherapy or in combination with riluzole. The neuroprotective effects of ATH-1105, which significantly slow neuromuscular deterioration in a relevant mouse model, may mitigate the progressive neurodegeneration that is a hallmark of ALS. Furthermore, the impact of ATH-1105 on reducing TDP-43 pathology and in normalizing the expression of autophagic proteins presents additional value for this approach by addressing pathophysiological components of ALS. Overall, the advancement of ATH-1105 as a therapeutic option for ALS holds substantial promise for restoring neuronal health in the face of a devastating neurodegenerative disease.

## Data Availability

The original contributions presented in the study are included in the article/Supplementary material, further inquiries can be directed to the corresponding author.

## Glossary

AlphaLISA: Amplified luminescent Proximity Homogenous Assay
ALS: Amyotrophic lateral sclerosis
ANOVA: Analysis of variance
BCA: Bicinchoninic acid
BDNF: Brain-derived neurotrophic factor
CFB: Change from baseline
CMAP: Compound muscle action potential
DMSO: Dimethyl sulfoxide
EDTA: Ethylenediaminetetraacetic acid
ELISA: Enzyme-linked immunosorbent assay
GAPDH: Glyceraldehyde-3-phosphate dehydrogenase
GSK3β: Glycogen synthase kinase-3-beta
HGF: Hepatocyte growth factor
HRP: Horseradish peroxide
i.p.: Intraperitoneally
IL-6: Interleukin-6
LAMP2: Lysosomal-associated membrane protein 2
LC3: Microtubule-associated protein 1A/1B-light chain 3
LSD: Least significant difference
MAP-2: Microtubule-associated protein 2
NCV: Nerve conduction velocity
NfL: Neurofilament light
NMJ: Neuromuscular junction
p.o.: Oral
PBS: Phosphate-buffered saline
PS: Penicillin–streptomycin
RT: Room temperature
SEM: Standard error of mean
siRNA: Small interfering RNA
SOD1: Superoxide dismutase 1
TDP-43: TAR DNA-binding protein 43
TNF-*α*: Tumor necrosis factor alpha
WT: Wild-type
