# Supplementary material for: ATH-1105 mitigates multiple pathologies in ALS models both alone and in combination with riluzole
Source: Front Neurol. 2026 Jan 8;16:1582765. doi: 10.3389/fneur.2025.1582765 (PMC12825224; doi:10.3389/fneur.2025.1582765)
Supplement: Supplementary file 1 [file Table_1.DOCX]

**
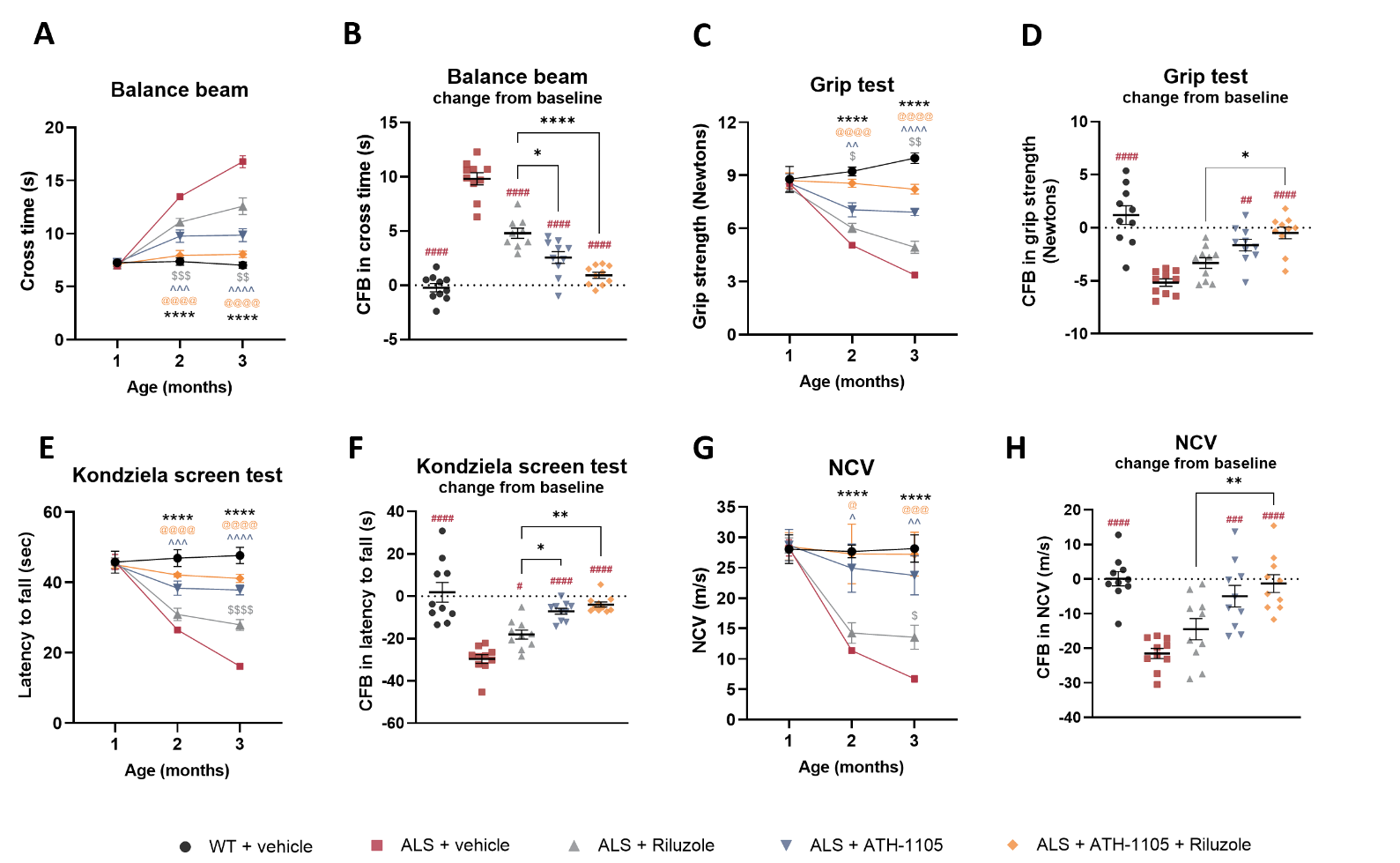
Supplementary Figure S1**

**Supplementary Figure S1. Additional measurements of motor and nerve function in Prp-TDP43^A315T^ ALS mice treated with ATH-1105, riluzole, or their combination.**

Evaluation of motor function via **(A)** balance beam cross time measured at baseline and after 1 and 2 months of treatment (at 1, 2, and 3 months of age), **(B)** balance beam cross time presented as change from baseline (CFB; from 1 to 3 months of age), **(C)** grip strength test over time, **(D)** CFB in grip strength, **(E)** kondziela screen test latency to fall over time, and **(F)** CFB in kondziela screen test performance. Nerve function was assessed via **(G)** nerve conduction velocity (NCV) in the sciatic nerve over time, and **(H)** CFB in NCV. Data are presented as mean ± SEM; n = 10. Statistical differences in panels A, C, E, and G were assessed using two-way ANOVA followed by Dunnett’s multiple comparisons test against ALS + vehicle. Symbols indicate comparisons to ALS + vehicle group as follows: “*” versus WT + vehicle, “$” versus ALS + Riluzole, “^” versus ALS + ATH-1105, and “@” versus ALS + ATH-1105 + Riluzole. For panels B, D, F, and H, one-way ANOVA followed by Tukey’s multiple comparisons test was used, where “#” represents comparisons versus ALS + vehicle and “*” represents all other significant comparisons as indicated. The following applies to all symbols: ^*^p<0.05, ^**^p<0.01, ^***^p<0.001, ^****^p<0.0001.

**
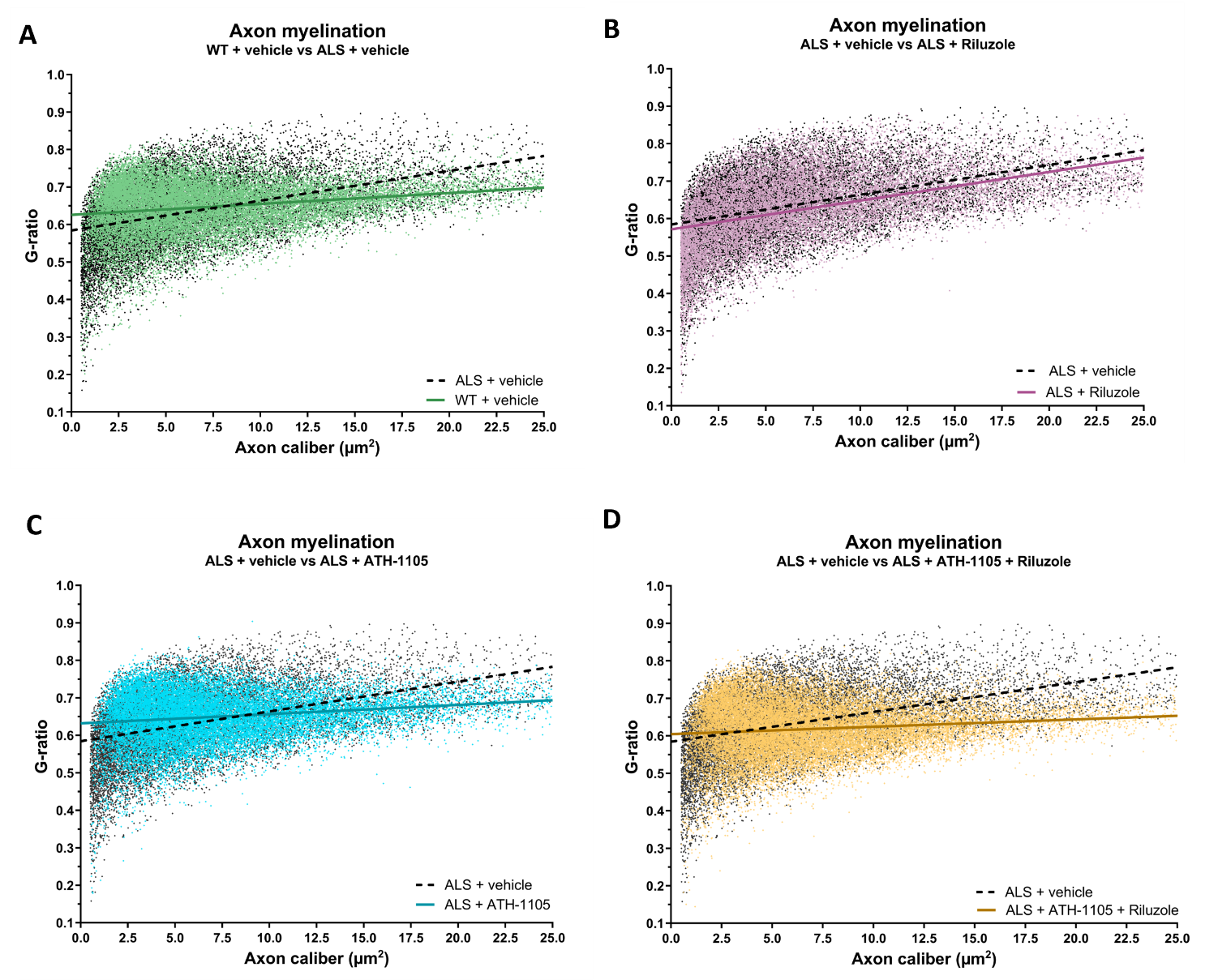
Supplementary Figure S2**

**Supplementary Figure S2. Individual datapoints used to generate axon caliber/g-ratio regression lines.**

Scatter plots demonstrating the relationship between axon caliber (µm^2^) and g-ratio (arbitrary units) in the sciatic nerve of WT or ALS mice after 2 months of respective treatments. Data are shown for the following groups: **(A)** WT + vehicle, **(B)** ALS + Riluzole, **(C)** ALS + ATH-1105, and **(D)** ALS + ATH-1105 + Riluzole, each superimposed on the ALS + vehicle group (black). Regression lines for each group are shown. Data points represent individual axons measured from sciatic nerves of n=10 mice per group.

**
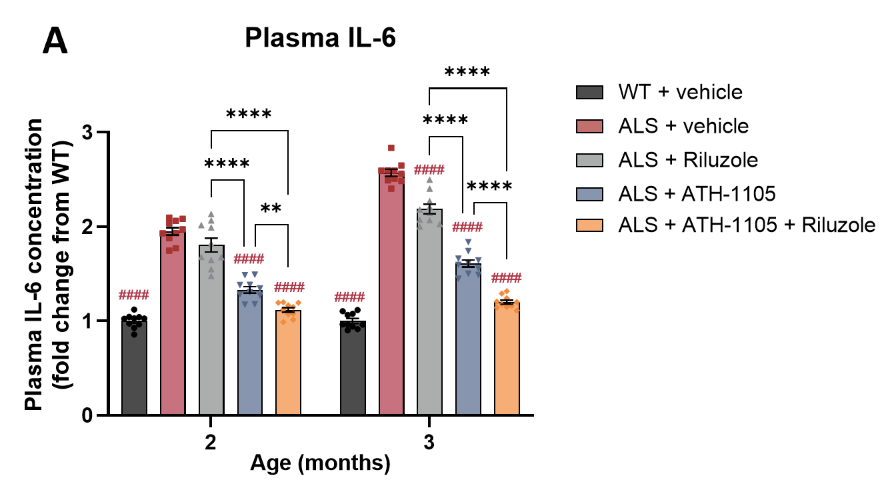
Supplementary Figure S3**

**Supplementary Figure S3. ATH-1105, riluzole, or their combination reduce plasma levels of IL-6 in ALS mice.**

**(A)** Graphical representation of IL-6 measured in the plasma of WT or ALS mice after 1 and 2 months of respective treatment (at 2 and 3 months of age), as a fold change in concentration from WT levels. Data are presented as mean ± SEM; n = 10. Statistical differences were determined by two-way ANOVA followed by Tukey’s multiple comparisons test, ^####^p<0.0001 versus ALS + vehicle, ^**^p<0.01 and ^****^p<0.0001 for the comparisons as indicated.

**Supplementary Figure S4**

**
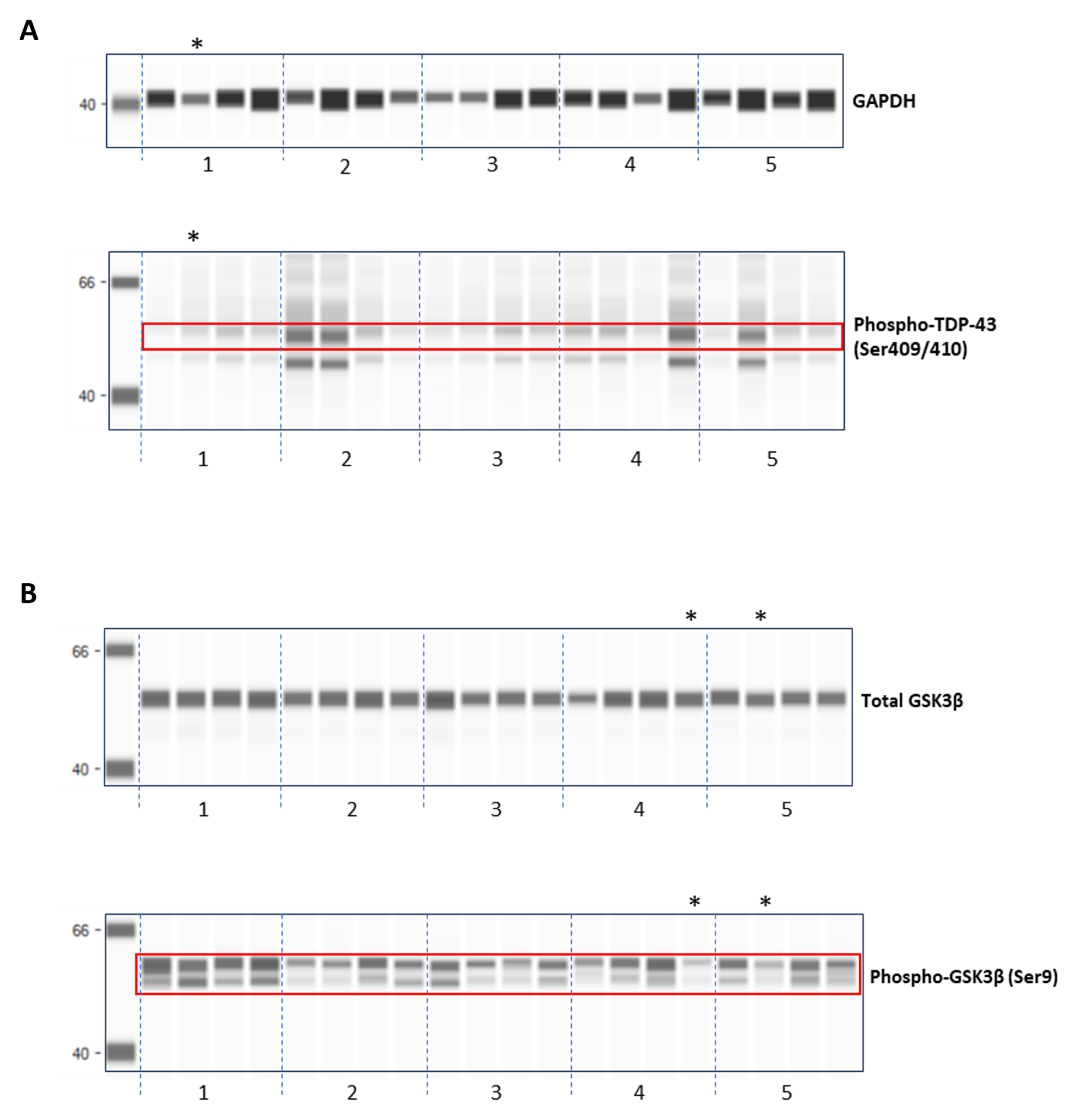
**

**Supplementary Figure S4. Representative western blot images showing effect of ATH-1105 on TDP-43 phosphorylation at Ser409/410 and GSK3β phosphorylation at Ser9**

Western blot images and analyses were obtained using the Simple Western™ system (Bio-Techne), an automated capillary-based immunoassay platform that performs protein separation, immunoprobing, detection, and quantitation in a fully automated manner. Primary rat spinal motor neurons were treated with ATH-1105 and glutamate (5 µM) for 24h and cell culture lysates were analyzed for **(A)** phoshpo-TDP-43(pTDP-43^Ser409/410^) and GAPDH, and **(B)** phospho-GSK3β (pGSK3β^Ser9^) and total GSK3β via Simple Western. Each protein sample was evaluated independently in a separate capillary system. Numbers correspond to four replicates per treatment: 1, control; 2, glutamate control; 3, glutamate + ATH-1105 (100 nM); 4, glutamate + ATH-1105 (500 nM); 5, glutamate + ATH-1105 (1 µM). Lanes labeled with ‘*’ indicate technical issues were identified during the assay or sample preparation and prior to quantification that prevented analysis from being successfully completed for that sample.

**Supplementary Figure S5**

**
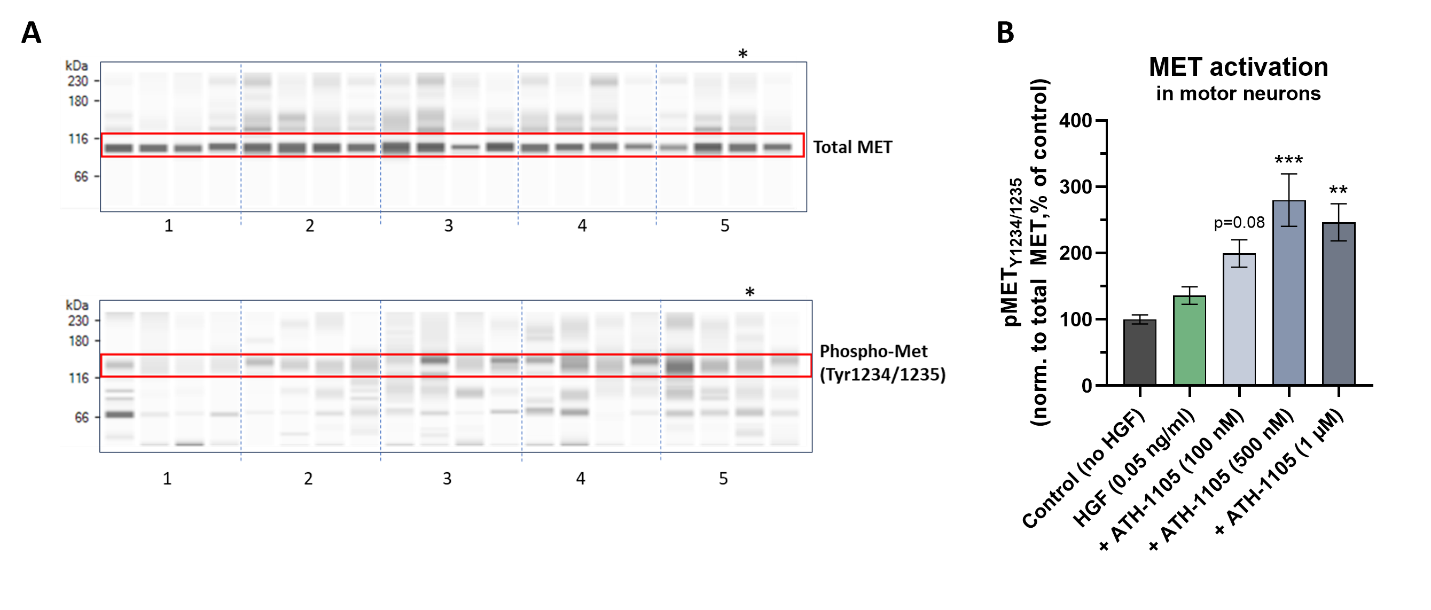
**

**Supplementary Figure S5. Effect of ATH-1105 on MET phosphorylation at Tyr1234/1235**

Western blot images and analyses were obtained using the Simple Western™ system (Bio-Techne), an automated capillary-based immunoassay platform that performs protein separation, immunoprobing, detection, and quantitation in a fully automated manner. Primary rat spinal motor neurons were exposed to a subthreshold level of exogenous HGF (0.05 ng/ml) in the absence or presence of ATH-1105 for 15 minutes and cell culture lysates were analyzed for **(A)** phospho-MET (pMET^Tyr1234/Tyr1235^) and total MET via Simple Western. Each protein sample was evaluated independently in a separate capillary system. Numbers correspond to four replicates per treatment: 1, control (no HGF); 2, HGF control (0.05 ng/ml); 3, HGF (0.05 ng/ml) + ATH-1105 (100 nM); 4, HGF (0.05 ng/ml) + ATH-1105 (500 nM); 5, HGF (0.05 ng/ml) + ATH-1105 (1 µM). Lanes labeled with ‘*’ indicate technical issues were identified during the assay or sample preparation and prior to quantification that prevented analysis from being successfully completed for that sample. **(B)** Quantification of MET phosphorylation (pMET/total MET) in cell culture lysates. Data are expressed as percentage of normal control (100%) and presented as mean ± SEM; n = 3-4 biological replicates. Statistical differences were determined by one-way ANOVA followed by Fisher’s LSD test versus HGF control. **p<0.01, ***p<0.001.


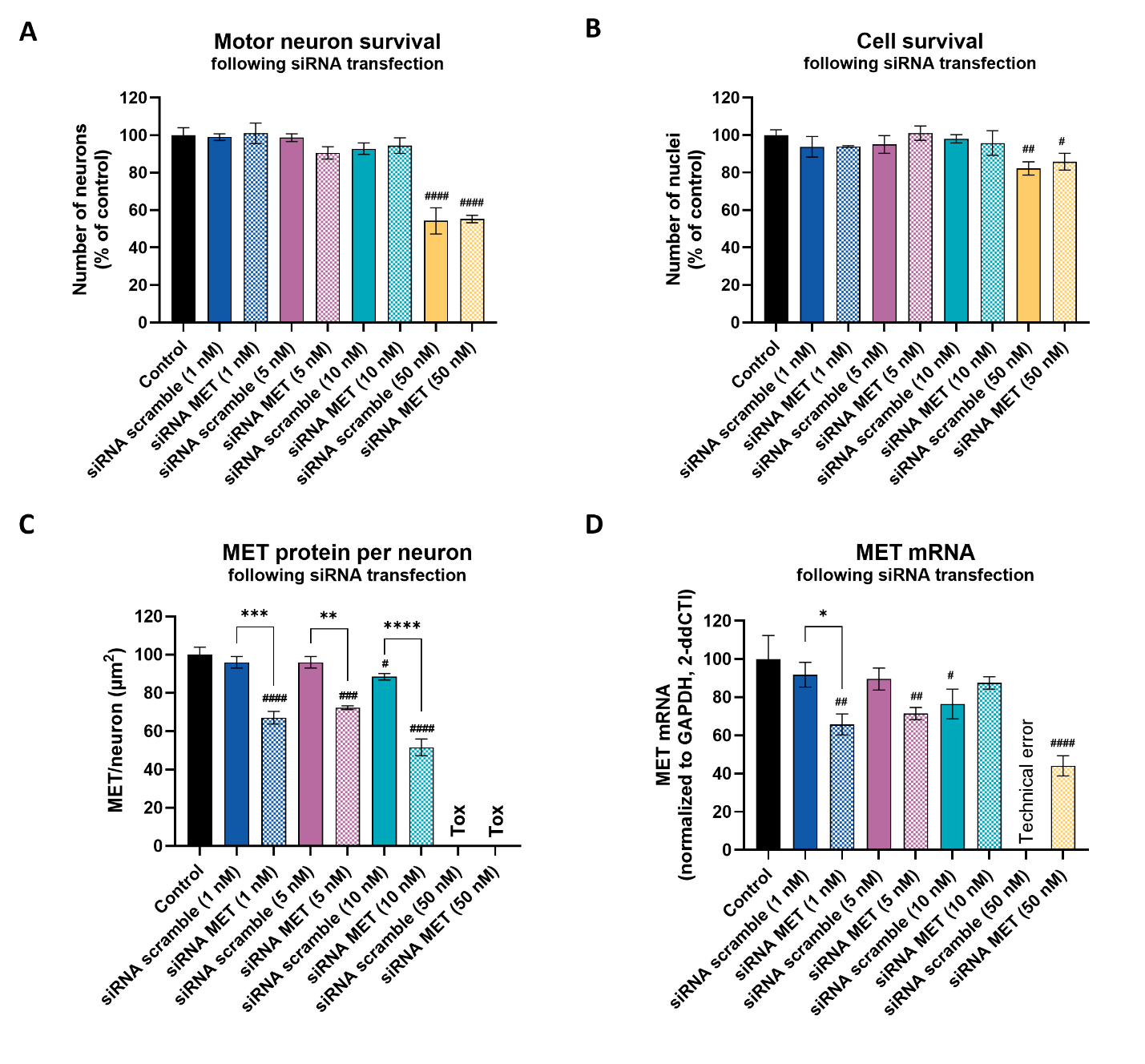
**Supplementary Figure S6**

**Supplementary Figure S6. MET receptor siRNA at 1 nM in primary motor neuron culture reduces MET protein and RNA expression without affecting neuron or cell numbers.**

Graphical representation of siRNA scrambled and siRNA c-MET, each at 1, 5, 10, and 50 nM, on (A) neuronal survival and (B) total cell number and on (C) c-MET area per neuron as detected by immunostaining and (D) c-MET mRNA expression as detected by qPCR. Data are expressed as percentage of normal control (100%) and presented as mean ± SEM; n = 3. Statistical differences were determined by one-way ANOVA followed by Fisher’s LSD test, where “#” indicates comparisons against control, and “*” for comparisons as bracketed. The following applies to all symbols: *p<0.05, **p<0.01, ***p<0.001, ****p<0.0001.
